# COPD lung studies of Nrf2 expression and the effects of Nrf2 activators

**DOI:** 10.1007/s10787-022-00967-3

**Published:** 2022-04-20

**Authors:** Jian Li, James Baker, Andrew Higham, Rajesh Shah, Angeles Montero-Fernandez, Clare Murray, Nicky Cooper, Cathy Lucas, Craig Fox, Dave Singh, Simon Lea

**Affiliations:** 1 Division of Immunology, Immunity to Infection and Respiratory Medicine, School of Biological Sciences, Faculty of Biology, Medicine and Health, Manchester Academic Health Science Centre, The University of Manchester and Manchester University NHS Foundation Trust, Manchester, UK; 2 2nd Floor Office Education and Research Centre, Wythenshawe Hospital, Southmoor Road, Manchester, M23 9LT UK; 3 Department of Thoracic Surgery, Manchester University Hospital NHS Foundation Trust, Manchester, UK; 4 Department of Histopathology, Manchester University Hospital NHS Foundation Trust, Manchester, UK; 5 C4X Discovery Ltd, Manchester, UK; 6 Medicines Evaluation Unit, Manchester University Hospital NHS Foundation Trust, The Langley Building, Southmoor Road, Manchester, UK

**Keywords:** Chronic obstructive pulmonary disease, Alveolar macrophages, Nrf2, KEAP1, Antioxidants

## Abstract

**Background:**

Nrf2 regulates cellular antioxidant defence in lung cells, including epithelial cells and alveolar macrophages (AM). The Nrf2/Keap-1 pathway can be modulated by activators with different modes of action; electrophilic compounds and protein–protein interaction (PPI) inhibitors.

We assessed Nrf2 and Keap-1 protein and gene levels in COPD compared to controls and the effect of Nrf2 activators on COPD AM.

**Methods:**

Lung resected tissue from non-smokers, smokers and COPD patients were analysed for epithelial and AM expression of Nrf2 and Keap-1 by imunoshistochemistry and by qPCR in isolated AM. AM were cultured with Nrf2 activators CDDO, C4X_6665, GSK7, MMF and Sulforaphane. Expression of Nrf2 target genes NQO1, HMOX1 SOD1 and TXNRD1 and NQO1 activity were assessed.

**Results:**

Nrf2 and Keap-1 expression was not altered in the epithelium or AM of COPD patients compared to controls. NQO1 activity was downregulated, while NQO1, HMOX1, SOD1 and TXNRD1 gene expression increased in COPD patients. All Nrf2 activators increased NQO1 activity, and NQO1, HMOX1, SOD1 and TXNRD1 expression in AMs from both COPD and smokers. The potency of C4X_6665 on NQO1 activity and regulation of Nrf2 target gene expression was higher than other compounds.

**Conclusion:**

There is evidence of dysregulation of the Nrf2 signalling pathway in AM from COPD patients**.** The higher potency of the novel PPI Nrf2 compound C4X_6665 for inducing antioxidant activity and gene expression compared to electrophilic and other PPI Nrf2 activators highlights the therapeutic potential of this compound to address Nrf2 pathway dysregulation in COPD AM.

**Supplementary Information:**

The online version contains supplementary material available at 10.1007/s10787-022-00967-3.

## Introduction

Chronic obstructive pulmonary disease (COPD) is characterised by airflow obstruction and persistent airway inflammation as a consequence of exposure to inhaled noxious particles, often from cigarette smoke (Vogelmeier et al. 2017). The presence of oxidants from cigarette smoke, other inhaled particles or red blood cells causes an imbalance between oxidant burden and antioxidant capacity in the lungs, leading to oxidative stress, inflammation and cell apoptosis (Kirkham and Barnes 2013; Marginean et al. 2018; Baker et al. 2021).

COPD involves the activation of a network of inflammation including epithelial cells, macrophages, neutrophils and lymphocytes (Hogg et al. 2004). Alveolar macrophages play a central role in co-ordinating the immune response in COPD, being capable of pro- and anti-inflammatory activity, as well as bacterial phagocytosis and efferocytosis (Vlahos and Bozinovski 2014). Macrophages show considerable plasticity, responding to environmental signals to alter their phenotype and function (Das et al. 2015). In COPD, there is evidence of skewing towards a dysfunctional alveolar macrophage phenotype with reduced efferocytosis and phagocytosis in response to the local microenvironment (Hodge et al. 2003; Harvey et al. 2011; Dewhurst et al. 2017).

Nuclear factor erythroid 2-related factor 2 (Nrf2, NFE2L2) is a major cell-intrinsic regulator of cytoprotective responses to oxidative stresses (Bellezza et al. 2018; Kansanen et al. 2013). Acting as a master transcription factor, it regulates the expression of various antioxidant genes including NAD(P)H dehydrogenase (quinone 1) (NQO1), heme-oxygenase (HO)-1 (HMOX1), thioredoxin reductase 1 (TXNRD1) and superoxide dismutase 1 (SOD1) via the antioxidant response element (ARE) signalling parthway (Nguyen et al. 2009; Jaramillo and Zhang 2013; Ma 2013). The repressor protein Kelch-like ECH-associated protein 1 (Keap-1) blocks Nrf2 dependent transcription under basal conditions. Oxidative stress causes Nrf2 nuclear translocation, thereby regulating target gene expression (Ma 2013). Animal and human studies have demonstrated that Nrf2/Keap-1 and their target genes protect against inflammation and oxidative stress from cigarette smoke (Kensler et al. 2007; Boutten et al. 2011), with Nrf2 disruption in mice leading to increased cigarette smoking-induced emphysema (Rangasamy et al. 2004; Cui et al. 2018). Furthermore, Nrf2 activators increase bacterial phagocytosis and killing in COPD macrophages, suggesting that this might be a useful pharmacological approach for COPD-associated defects in bacterial clearance (Bewley et al. 2018).

In humans, it has been reported that current smoking increased mRNA expression of NFE2L2 and associated genes including NQO1 and HMOX1in bronchial epithelium (Sidhaye et al. 2019), while NFE2L2 mRNA expression and associated genes were increased in PBMCs from mild COPD patients (Fratta Pasini et al. 2016, 2020). In contrast, other studies have reported that NFE2L2 mRNA expression was reduced in alveolar macrophages and bronchial epithelium from mild COPD patients compared to controls (Suzuki et al. 2008) and Nrf2 protein and mRNA expression were reduced in alveolar macrophages from severe COPD patients (Goven et al. 2008; Fratta Pasini et al. 2020). These conflicting data, showing both up- and down-regulation of Nrf2 expression, have concentrated mainly on gene expression analysis; there is less information regarding protein expression and downstream functional activity.

The Nrf2/Keap-1 pathway can be modulated by activators that work by different modes of action (Kwak and Kensler 2010; Rojo de la Vega et al. 2016). Electrophilic compounds such as the synthetic triterpenoid 2-cyano-3,12-dioxooleana-1,9(11)-dien-28-oic acid (CDDO), Sulforaphane and monomethylfumarate (MMF, the active metabolite of dimethylfumarate) activate Nrf2 through irreversible covalent modification of cysteine residues on Keap-1 (Satoh and Lipton 2017; Cleasby et al. 2014). However, these compounds can also modify cysteine residues on other proteins, potentially causing unwanted, off-target pharmacological activity. Keap-1-Nrf2 protein–protein interaction (PPI) inhibitors are non-covalent, small synthetic compounds that bind to Keap-1 and block the protein–protein interaction between Keap-1 and Nrf2 (Liu et al. 2019). These PPI inhibitors, which include GSK compound 7 (GSK7) (Davies et al. 2016) and a proprietary compound C4X_6665 covered by a patent filed by C4X Discovery (WO2020/084300), offer a potentially safer alternative since they are designed to be highly specific and reversible.

As previous studies have reported conflicting NFE2L2 gene expression data in COPD compared to controls (Fratta Pasini et al. 2016, 2020; Goven et al. 2008; Suzuki et al. 2008), we conducted protein expression studies for Nrf2 and Keap-1 in alveolar macrophages and the bronchial epithelium, using lung tissue from COPD patients compared to control non-smokers (NS) and smokers (S). We also investigated the effects of different Nrf2 activators on NQO1 activity and expression of Nrf2 target genes (NQO1, HMOX1, SOD1 and TXNRD1) in COPD alveolar macrophages, focusing on the comparison of the electrophilic compound CDDO with the PPI inhibitors GSK7 and C4X_6665.

## Methods

### Study subjects

One hundred and twenty-one patients undergoing surgical resection for suspected lung cancer were recruited (20 NS, 45 S and 56 COPD patients; overall demographics in Table 1, and in supplement for each experiment Tables S1–3). COPD was diagnosed based on GOLD recommendations (Vogelmeier et al. 2017). Controls were smokers (S) without airflow limitation, or non-smokers (NS) (pack-year history < 1). This research was approved by the relevant local research ethics committees (reference 03/SM/396–South Manchester Ethics Committee and 20/NW/0302—North West Ethics Committee) and all experiments were performed in accordance with relevant guidelines and regulations. All subjects provided written informed consent.

**Table 1:** Demographics of the study population

	Never smoker	Smokers	COPD	*p* value
*n*	20	45	56	N/A
Age (y)	69 (11)	66 (8)	67 (8)	*p *> 0.05
Gender: male (%)	20	38	47	*p *> 0.05
FEV_1_ (L)	2.3 (0.8)	2.3 (0.5)	1.7 (0.5)*^†^	*p *< 0.001
FEV_1_% predicted	110 (24)	99 (18)	68 (15)*^†^	*p *< 0.001
FVC (L)	2.9 (1.0)	3.1 (0.7)	3.1 (0.7)	*p *> 0.05
FEV_1_/FVC ratio (%)	83 (8)	79 (10)	51 (16)*^†^	*p *< 0.001
Current smokers (%)	N/A	78	65	*p *> 0.05
Pack year history	N/A	39 (16)	51 (36)	*p *> 0.05
ICS users (%)	N/A	4	27	N/A
LABA users (%)	N/A	2	33	N/A
LAMA users (%)	N/A	N/A	24	N/A

FEV_1_ forced expiratory volume in 1 s; FVC forced vital capacity; ICS inhaled corticosteroids. LABA long-acting beta-2 agonists; LAMA long-acting muscarinic antagonists Data presented as mean (SD). Tukey’s multiple comparisons test performed where

*Significant difference between COPD vs NS

^†^Significant difference between COPD vs S

### Immunohistochemistry

Tissue blocks were obtained from an area of the lung as far distal to the tumour as possible and processed as previously described (Lea et al. 2014). Blocks were labelled using anti-Nrf2 and anti-Keap-1 primary antibodies. Lung tissue was fixed in 10% neutral buffered formalin for 8 h and processed using an automated tissue processing machine on a routine overnight schedule. Biopsies were embedded in histological grade paraffin wax and 3 µm sections were cut with a Leica RM2235 rotary microtome. Sections were stained with anti-Nrf2 and anti-Keap-1 (Abcam) overnight at 4 °C coupled with an ImmPRESS^™^ Excel Amplified HRP Polymer Staining Kit (Anti-Rabbit IgG) with 3,3′ diaminobenzidine as a substrate (Vector Laboratories, CA, USA). Full IHC procedure was described before (Pergola et al. 2011). Briefly, slides were dewaxed using xylene and dehydrated through a series of industrial denatured alcohols. Heat-induced epitope retrieval was achieved using citrate buffer (pH 6.0) and a microwave. Sections were counterstained in Gills haematoxylin. A rabbit IgG isotype, diluted to the same concentration as the relevant primary antibody was used as a negative control (Cell Signaling Technology). Following IHC, images (4 per slide) were captured using a Nikon Eclipse 80i microscope (Nikon UK Ltd) with an attached QImaging digital camera (Media Cybernetics, MD, USA). Percentages of Nrf2 or Keap-1-positive epithelial cells and macrophages were calculated using the cell counting tool in ImageJ (version 1.49, NIH). The percentages of positive alveolar macrophages of each biopsy were calculated within the alveolar space. At least 200 macrophages, defined as mononuclear cells with well-represented cytoplasm present in the alveolar spaces and not attached to the alveolar walls, were counted and the number of positively stained cells was expressed as a percentage (Lea et al. 2014). In a subset of patients with small airways present on the stained section protein expression of Nrf2 and Keap-1 was examined by immunohistochemistry in lung tissue and the number of positively stained cells was expressed as a percentage of the total number of epithelial cells.

### Lung macrophages isolation, culture and compound treatments

Alveolar macrophages from resected lung tissue from COPD patients and controls were isolated as previously described (Higham et al. 2014). Lung tissue was obtained through surgery from patients with suspected cancer. Tissue was then taken from areas most distal from the tumour. Resected lung tissue airways were perfused with 0.1 M NaCl. The subsequent fluid was then centrifuged for 10 min at 400 g at room temperature. The cell pellet was then resuspended in RPMI-1640 media (Sigma-Aldrich, Poole, UK). The cell suspension was then layered over Ficoll-Paque (GE Healthcare, Buckinghamshire, UK) and was centrifuged at 400 g for 30 min at room temperature with the no-brake setting. The interphase was removed from the Ficoll suspension using a pasture pipette and was washed with RMPI-1640 with 1% Penicillin Streptomycin Solution, 1% L-Glutamine and 10% Fetal Calf Serum. Cell counting was performed using a haemocytometer (Neubauer haemocytometer) and Trypan Blue exclusion dye and cells plated for culture at a density of 1 × 10^6^ per ml. Cells were cultured overnight before non-adherent cells removed. Following isolation, macrophages were cultured (37 °C, 5% CO_2_) with and without Nrf2 activators at a range of doses for 24 or 48 h depending on experiments. Nrf2 activator compounds used were: CDDO (Bardoxolone/CDDO-Methyl) (Cayman Chemicals) (0.3–100 nM), GSK7 (C4X Discovery Ltd) (1–1000 nM), MMF (C4X Discovery Ltd) (1000–10,000 nM), Sulforaphane (a naturally occurring isothiocyanate) (Sigma Aldrich) (1000–10,000 nM) and C4X_6665 (C4X Discovery Ltd) (0.3–30 nM). Compound concentration ranges were chosen based on previously published data for CDDO and GSK7 (Davies et al. 2016), Sulforaphane (Bewley et al. 2018), MMF (Ahuja et al. 2016) and on preliminary data for C4X_6665 (Supplement Fig. 1). A greater range of concentrations was used for CDDO, GSK7 and C4X_6665 to better compare concentration–response curves. All compounds were dissolved in DMSO (0.01% final in assay), which was used as vehicle control. There was no cytotoxicity observed for any Nrf2 activator compound at all concentrations (Supplement Fig. 2). Representative images of isolated macrophages treated with either CDDO (100 nM), C4X_6665 (30 nM), GSK7 (1000 nM), Sulforaphane (10,000 nM), MMF (10,000 nM) or DMSO control for 24 h are shown in the online supplement (Supplement Fig. 3).

Cells were harvested for analysis of NQO1 activity at 48 h (previously validated as the optimal time point for this method (Davies et al. 2016)), expression of NQO1, HMOX1, SOD1 and TXNRD1 analysed by qPCR at 24 h and cell viability using a lactate dehydrogenase (LDH) assay at 48 h.

### Quantitative PCR

Culture supernatants were removed and cells were lysed in RLT buffer. Total RNA was purified from cell lysates using RNeasy kits (Qiagen, Crawley, U.K.) according to the manufacturer’s instructions. DNA contamination was prevented by on-column addition of DNase (Qiagen, Crawley, U.K.) according to the manufacturer’s instructions. Reverse transcription was performed on 50 ng of RNA using the Verso cDNA kit (Thermo Scientific). The resulting cDNA was reacted with ABsolute blue qPCR mix (Thermo Scientific) in 25 µl reactions containing premade ABI Taqman gene expression assays for NFE2L2, KEAP1, NQO1, HMOX1, SOD1 and TXNRD1 and the endogenous control was glyceraldehyde-3-phosphate dehydrogenase (GAPDH) (catalogue no.: 4352934E) (Applied Biosystems, Warrington, U.K.). Controls without RT-enzyme showed there was no genomic DNA amplification. Thermal cycling was carried out on a Stratagene MX3005P (Agilent Technologies, West Lothian, U.K.). Relative expression levels were determined using the 2^−ΔΔ^*C*_t_ (Nrf2 activator treatment fold change relative to DMSO control) or 2^−Δ^*C*_t_ (relative to endogenous control for baseline expression and maximal expression induction).

### NQO1 activity enzyme assay

NQO1 is a homodimeric FAD-containing enzyme that catalyzes obligatory NAD(P)H-dependent two-electron reductions of quinones and protects cells against the toxic and neoplastic effects of free radicals and reactive oxygen species. The transcription of NQO1 is highly inducible by Nrf2, and thus NQO1 activity is a faithful marker for Nrf2 activation. Macrophage cells were seeded in 96 well black clear-bottomed plates at a concentration of 10^5^ cells/well in 100 μl of supplemented RPMI medium (5% FBS with 2 mM glutamine, penicillin and streptomycin) and incubated at 37 °C, 5% CO_2_ overnight. Media was changed and compounds or DMSO control (0.01% final in assay) were added to the cells in 200 μl final volume incubated at 37 °C, 5% CO_2_ for 48 h. Medium was aspirated from the plate and crude cell lysates were made by adding 50uµl of 1X Cell Signaling Technologies lysis buffer with 1 Complete, Mini, EDTA-free Protease Inhibitor Tablet (Roche) for each 10 ml of lysis buffer. After lysis plates were incubated for 20 min at room temperature, 50 μl of MTT cocktail was added to each well and analysed on POLAstar Omega (BMG LABTECH) using Absorbance 570 nm for 0 and 30 min.

### Cytotoxicity assay-LDH assay

The Pierce LDH Cytotoxicity Assay Kit (Thermo Scientific) was used to measure cytotoxicity mediated by Nrf2 compounds. 10^5^ cells/well were seeded in 96 well plate in 200 μl RPMI medium (5% FBS with 2 mM glutamine, penicillin and streptomycin) with compounds or DMSO control (0.01% final in assay) and incubated at 37 °C, 5% CO_2_ for 48 h. 50 μl of supernatant was transferred into a new plate and mixed with reaction buffer. After 30 min of incubation at room temperature, reactions were stopped by adding Stop Solution. Absorbance at 490 nm and 680 nm was measured using a plate-reading spectrophotometer to determine LDH activity.

### Data analysis

One-way ANOVA followed by Tukey’s test was used for IHC data to compare between subject groups. Maximal effect was defined as the effect at the highest concentration used for each compound. Two-way analysis of variance (ANOVA) followed by Tukey’s multiple comparisons test was used for compound treatment experiments to compare responses of smokers and COPD patients to different doses of compounds, and whether there is an interaction between drug concentrations. EC_50_ (50% maximal efficacy concentration) was determined for CDDO, GSK7 and C4X_6665 for individual compounds. Data were log transformed and normalised by setting the maximal effect to 100% before using a non-linear iterative curve fitting analysis to generate EC_50_. All statistical analysis was performed in GraphPad Prism (GraphPad Software, http://www.graphpad.com). *p *< 0.05 was considered significant.

## Results

### Nrf2 and Keap-1 protein and gene expression

**Fig. 1:**
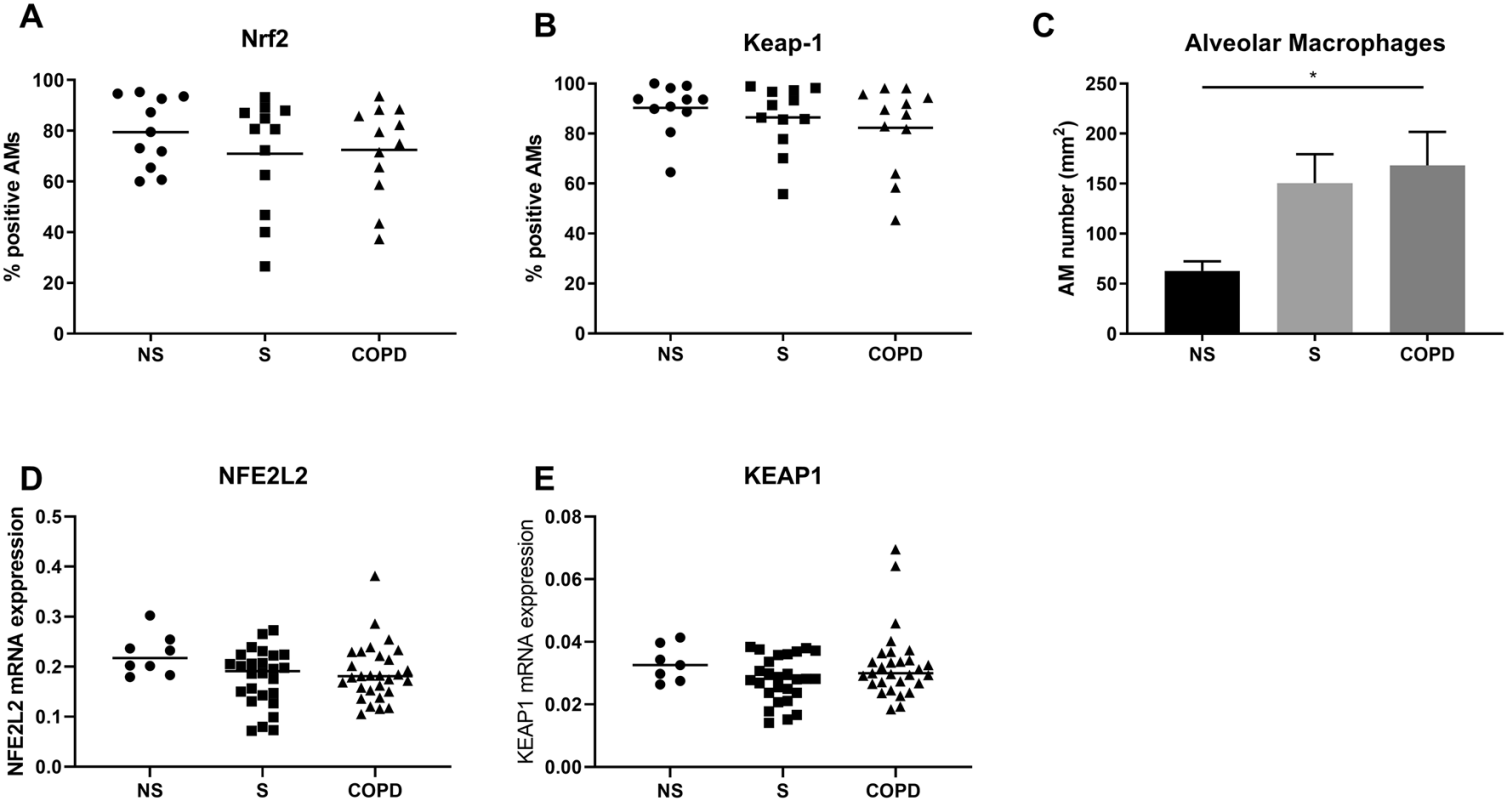
Expression of Nrf2 and Keap-1 in the alveolar macrophages of NS, S and COPD patients. Protein expression of Nrf2 and Keap-1 was examined by immunohistochemistry in lung tissue from NS (*n *= 12), S (*n *= 12) and COPD (*n *= 12). Data presented as percentage of positive cells of alveolar macrophages (AM) for Nrf2 (**A**) and Keap-1 (**B**). The number of alveolar macrophages per area of airways in lung tissue was determined (**C**). mRNA expression levels of NFE2L2 (**D**) and KEAP1 (**E**) were determined by RT-qPCR in AM from NS (*n *= 8), S (*n *= 25) and COPD (*n *= 29). Data represent individual patients with mean. RT-qPCR data expression relative to an endogenous control (2^−Δ^*C*_t_)

Immunohistochemistry was performed to examine Nrf2 and Keap-1 protein expression in alveolar macrophages and bronchial epithelium of NS (*n *= 12), S (*n *= 12) and COPD patients (*n *= 12) (Supplement Figs. 4 and 5). There was no significant difference in the percentage of alveolar macrophages expressing Nrf2 or Keap-1 protein between groups (Fig. 1A and B, respectively). The number of alveolar macrophages was significantly greater in COPD patients compared to NS (Fig. 1C *p* < 0.05). The mRNA expression levels of NFE2L2 and KEAP1 in alveolar macrophages from NS (*n *= 8), S (*n *= 25) and COPD patients (*n *= 29) were similar (Fig. 1D and E, respectively).

Sub-analysis of the COPD group showed no significant differences in Nrf2 or Keap-1 protein or mRNA expression levels between those patients taking inhaled corticosteroids and/or long-acting bronchodilator treatment versus those without these treatments (Supplement Fig. 6).

**Fig. 2:**
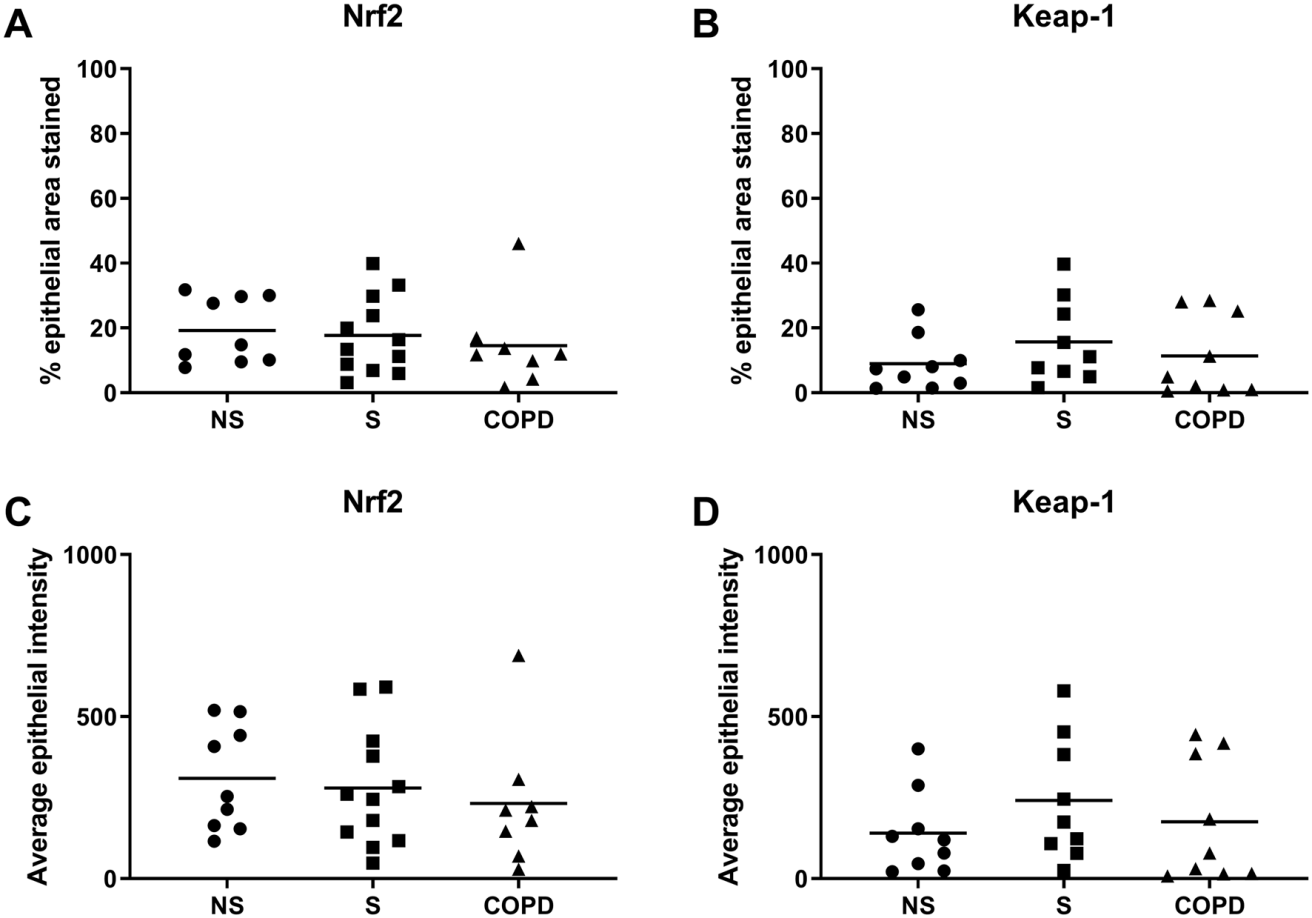
Expression of Nrf2 and Keap-1 in the bronchial epithelium of NS, S and COPD patients. Protein expression of Nrf2 and Keap-1 was examined by immunohistochemistry in lung tissue from NS (*n *= 9), S (*n *= 12) and COPD (*n *= 8). Data presented as positive cells per mm of epithelium for Nrf2 (**A**) and Keap-1 (**B**). Data represented as positive cells intensity of epithelium for Nrf2 (**C**) and Keap-1 (**D**). Data represent individual patients with mean

There were no significant differences between groups in the percentage of epithelium immunoreactive for Nrf2 or Keap-1 (Fig. 2A and B, respectively), nor the intensity of Nrf2 or Keap-1 staining (Fig. 2C and D, respectively). Also sub-analysis of the COPD group showed no significant differences in Nrf2 or Keap-1 protein expression levels between those patients taking inhaled corticosteroids and/or long-acting bronchodilator treatment versus those without these treatments (Supplement Fig. 7).

### NQO1 activity and antioxidant gene expression in COPD patients and control macrophages

**Fig. 3:**
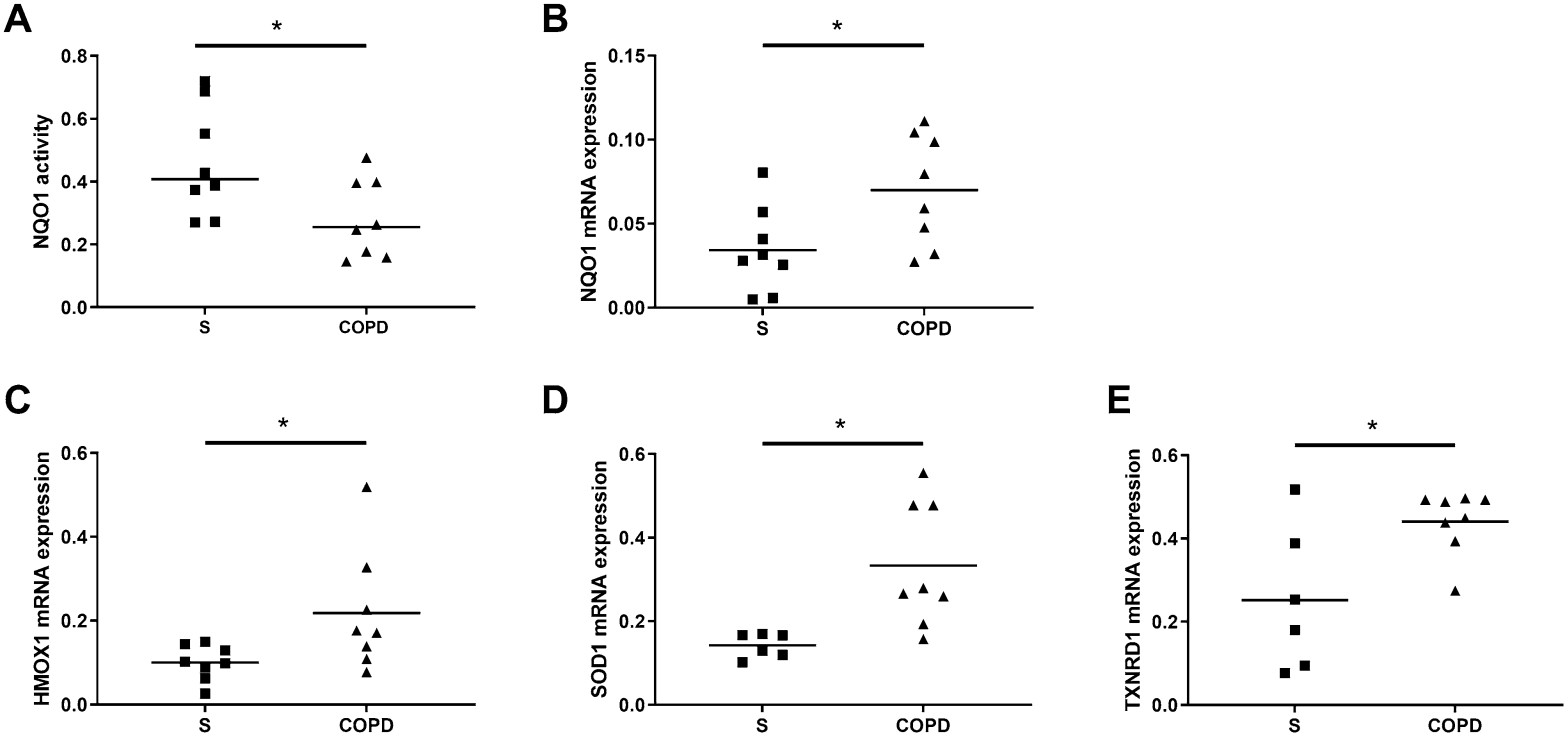
Basal NQO1 activity, NQO1, HMOX1, SOD1 and TXNRD1 mRNA expression in alveolar macrophages from S and COPD patients. Alveolar macrophages were isolated from S and COPD patients. Basal levels of NQO1 activity were assessed by NQO1 activity enzyme assay (**A**) (*n *= 8 S and *n *= 8 COPD). Basal mRNA expression of NQO1 (*n *= 8 S and *n *= 8 COPD), HMOX1 (*n *= 8 S and *n *= 8 COPD), SOD1 (*n *= 6 S and *n *= 8 COPD) and TXNRD1 (*n *= 6 S and *n *= 8 COPD) was assessed by RT-qPCR relative to endogenous control (2^−Δ^*C*_t_) (**B**–**E** respectively). Data represent individual patients with mean. * = significant difference between groups (*p *< 0.05)

The levels of NQO1 activity were significantly lower in alveolar macrophages from COPD patients (*n *= 8) compared to S (*n *= 8) (Fig. 3A *p* < 0.05). The mRNA levels of NQO1, HMOX1, SOD1 and TXNRD1 were significantly higher in alveolar macrophages from COPD patients compared to S (Fig. 3B–E, respectively, *p *< 0.05).

### Effect of Nrf2 activators on macrophage NQO1 expression and NQO1 activity

**Fig. 4:**
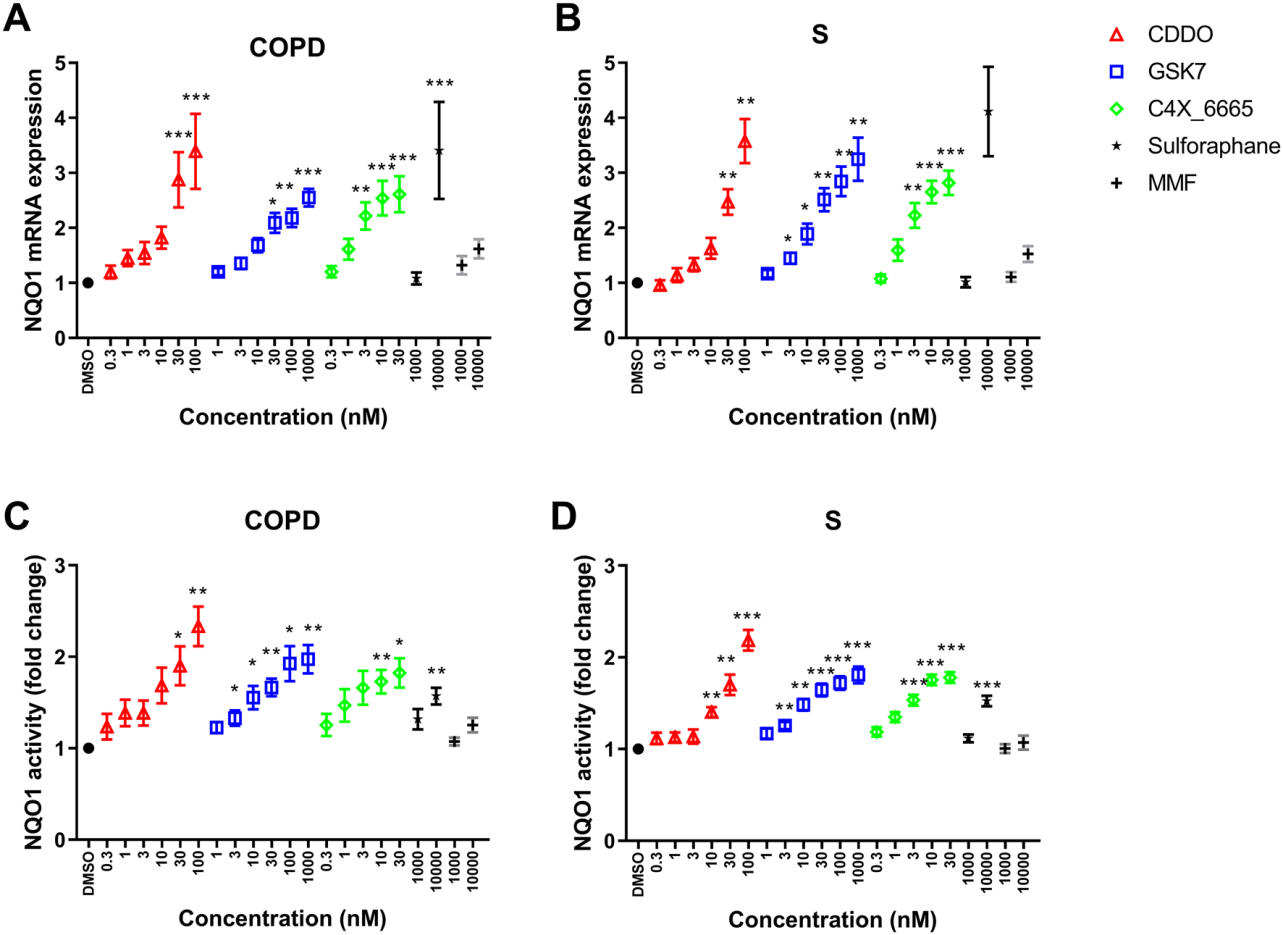
Effect of Nrf2 activator compounds on NQO1 mRNA expression and NQO1 activity in alveolar macrophages from S and COPD patients. Alveolar macrophages from COPD patients (*n *= 8) (**A** and **C**) and S (*n *= 8) (**B** and **D**) were treated with CDDO (0.3–100 nM), GSK7 (1–1000 nM), C4X_6665 (0.3–30 nM), Sulforaphane (1000–10,000 nM), MMF (1000–10,000 nM) or vehicle control (DMSO) for 24 h for qPCR (**A**, **B**) or 48 h for activity assay (**C**, **D**). NQO1 mRNA expression was assessed by RT-qPCR (**A**, **B**) and NQO1 activity was assessed by NQO1 activity enzyme assay (**C**, **D**). Data presented as mean ± SEM fold increase above DMSO control (RT-qPCR data 2^−ΔΔ^*C*_t_). *, **, *** = significantly above DMSO control (*p *< 0.05, *p *< 0.01 and *p *< 0.001, respectively)

Alveolar macrophages from COPD patients (*n *= 8) and S (*n *= 8) were treated with the Nrf2 activators CDDO, GSK7, C4X_6665, Sulforaphane and MMF. NQO1 mRNA expression levels were determined after 24 h, while NQO1 activity was assessed after 48 h. Nrf2 activators caused significant dose-dependent increases of NQO1 mRNA expression and NQO1 activity in alveolar macrophages from COPD patients (Fig. 4A and C, respectively) and S (Fig. 4B and D, respectively). In the NQO1 activity assay, CDDO (100 nM) had the greatest maximal effects (the effect at the highest concentration), followed by GSK7 (1000 nM) and C4X_6665 (30 nM), then Sulforaphane (10,000 nM), with MMF (10,000 nM) having the lowest activity (Table 2). The maximal effects for MMF were lower than the other drugs for NQO1 expression in COPD and S (Table 2).

**Table 2:** Maximum effect and EC_50_s of Nrf2 activator compounds on alveolar macrophage NQO1 mRNA expression and NQO1 activity

	S (*n *= 8)			COPD (*n* = 8)		
	Maximum effect (fold increase)		EC_50_ (nM)	Maximum effect (fold increase)		EC_50_ (nM)
NQO1 mRNA expression						
CDDO	3.58^††^	19.7		3.39	11.8	
GSK compound 7	3.25^††^	13.1		2.55^†††^	13.9	
C4X_6665	2.82^††^	1.9		2.61^†^	1.5	
Sulforaphane	4.12	–		3.41	–	
MMF	1.53	–		1.62	–	
ANOVA *p* value	0.0087	–		0.065		
NQO1 activity						
CDDO	2.19**^$$¶¶†††^	15.3		2.33*^$$¶¶††^	7.3	
GSK compound 7	1.81^†^	6.1		1.97^¶††^	8.9	
C4X_6665	1.78^¶††^	1.3		1.82^††^	1.2	
Sulforaphane	1.52^††^	–		1.57^†††^	–	
MMF	1.07	–		1.25	–	
ANOVA *p* value	< 0.0001			< 0.0001		

*, **Significantly above GSK7 (*p *< 0.05, 0.01)

^$, $$^Significantly above C4X_6665 (*p *< 0.05, 0.01)

^¶, ¶¶^Significantly above Sulforaphane (*p *< 0.05, 0.01)

^†, ††, †††^Significantly above MMF (*p *< 0.05, 0.01, 0.001)

**Fig. 5:**
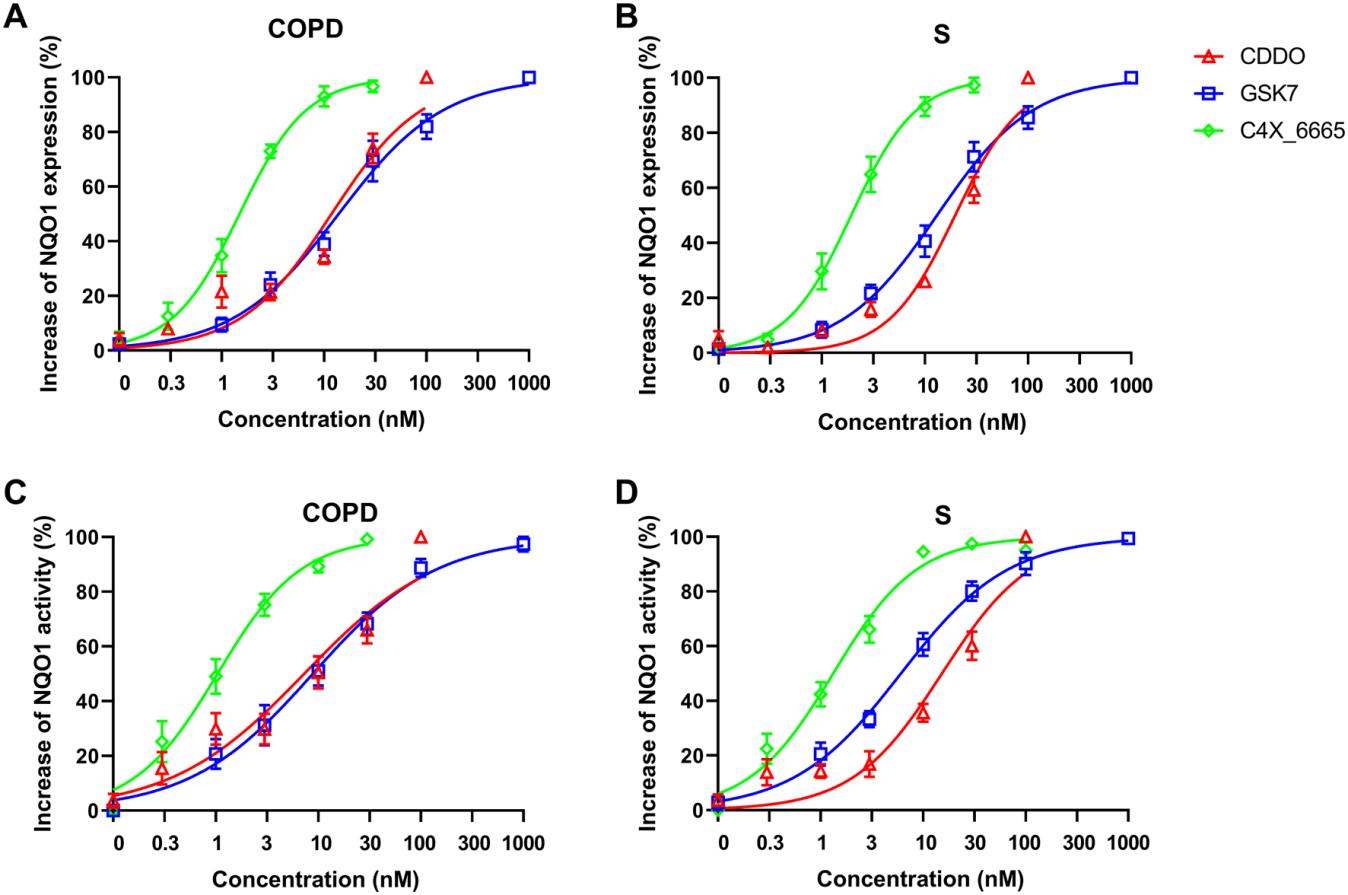
Effect of Nrf2 activator compounds on NQO1 mRNA expression and NQO1 activity in alveolar macrophages from S and COPD patients. Alveolar macrophages from COPD patients (*n *= 8) (**A** and **C**) and S (*n *= 8) (**B** and **D**) were treated with CDDO (0.3–100 nM), GSK7 (1–1000 nM), C4X_6665 (0.3–30 nM), or vehicle control (DMSO) for 24 h for qPCR (**A**, **B**) or 48 h for activity assay (**C**, **D**). NQO1 mRNA expression was assessed by RT-qPCR (**A**, **B**) and NQO1 activity was assessed by NQO1 activity enzyme assay (**C**, **D**). Four parameters non-linear iterative curve fitting analysis for individual compounds was used to generate curves

The effect at matched concentrations was compared between CDDO, GSK7 and C4X_6665 (Table S4). C4X_6665 had a greater effect on both NQO1 mRNA expression and activity compared to GSK7 and CDDO (at 3–10 nM) in both groups. EC_50_ values for NQO1 mRNA expression and activity (Fig. 5A and C, respectively) were lower for C4X_6665 (1.5 and 1.2 nM, respectively) compared to CDDO (11.8.1 and 7.3 nM, respectively) and GSK7 (13.9 and 8.9 nM, respectively) in COPD patients (Table 2). Similar results were observed in S (Fig. 5B and D and Table 2).

There were no differences between COPD patients or S for the change (difference between baseline and maximal effect) in NQO1 mRNA expression or activity induced by any compound. Further details are in the online supplement.

### Effect of Nrf2 activators on HMOX1, SOD1 and TXNRD1 expression in macrophages

**Fig. 6:**
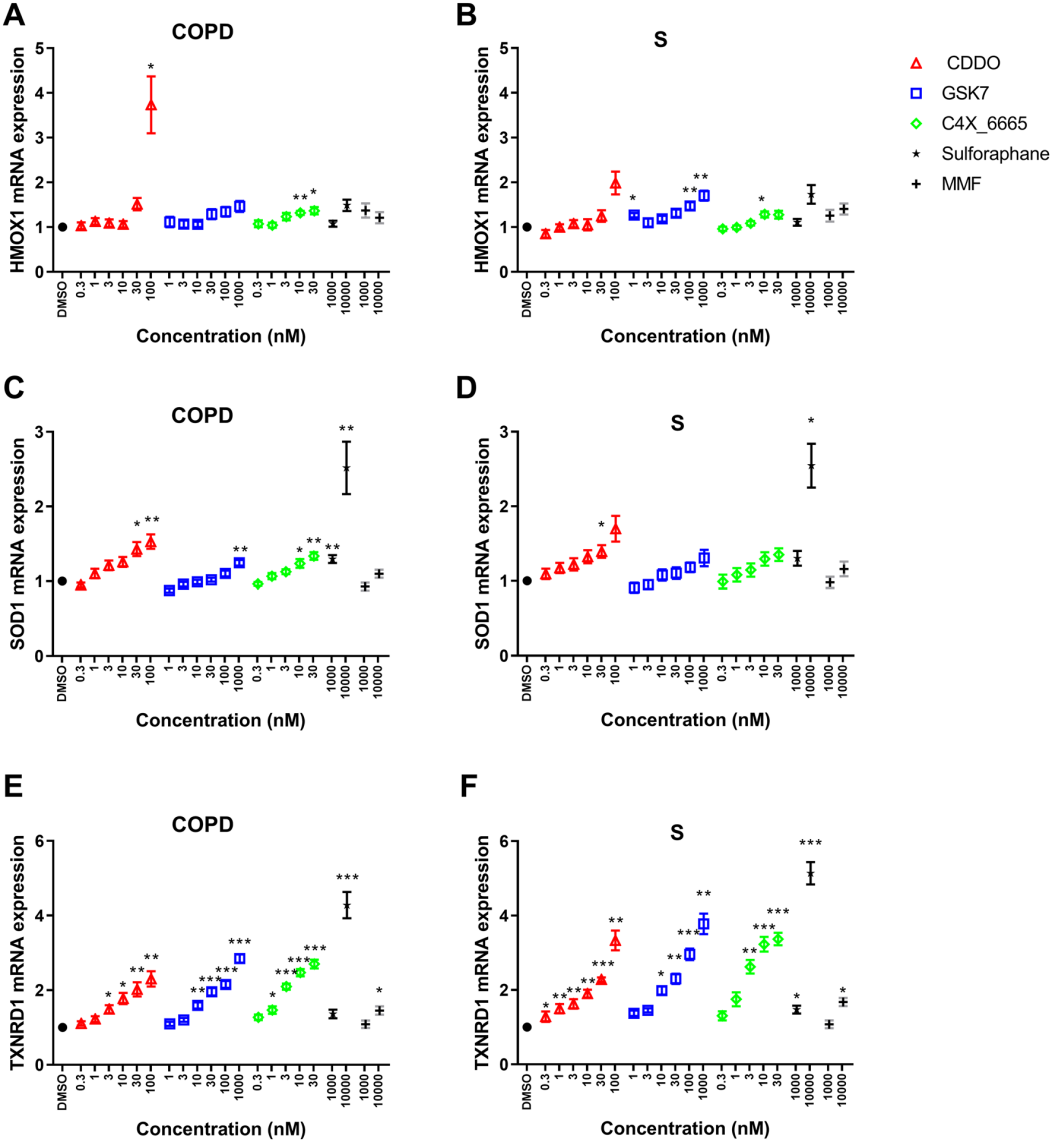
Effect of Nrf2 activator compounds on HMOX1, SOD1 and TXNRD1 mRNA expression in alveolar macrophage from S and COPD patients. Alveolar macrophages from COPD patients and S were treated with CDDO (0.3–100 nM), GSK7 (1–10,00 nM), C4X_6665 (0.3–30 nM), Sulforaphane (1000–10,000 nM), MMF 1000–10,000 nM) or vehicle control (DMSO) for 24 h for qPCR. HMOX1 (*n *= 8 COPD and *n *= 8 S) (**A** and **B**, respectively), SOD1 (*n *= 8 COPD and *n *= 6 S) (**C** and **D**, respectively) and TXNRD1 (*n *= 8 COPD and *n *= 6 S) (**E** and **F**, respectively) mRNA expression was assessed by RT-qPCR. Data presented as mean ± SEM fold increase above DMSO control (RT-qPCR data 2^−ΔΔ^*C*_t_). *, **, *** = significantly above DMSO control (*p *< 0.05, *p *< 0.01 and *p *< 0.001, respectively)

Alveolar macrophages from S (*n *= 8) and COPD patients (*n *= 8) were treated with Nrf2 activators for 24 h and mRNA expression levels of HMOX1, SOD1 and TXNRD1 were measured. The magnitude of upregulation of HMOX1 and SOD1 was generally < twofold, while TXNRD1 upregulation was greater. There was significant upregulation (*p *< 0.05) of TXNRD1 by all Nrf2 activators in both groups (Fig. 6E–F). For HMOX1 and SOD1, significant upregulation was less consistently achieved by Nrf2 activators, although all except MMF demonstrated significant upregulation of SOD1 in COPD patients. The EC_50_ values for upregulation of TXNRD1 and SOD1 were 2.0 and 3.1 nM, respectively, for C4X_6665 in COPD patients, with higher values for CDDO (5.7 and 4.8 nM, respectively) and GSK7 (30.4 and 31.9 nM, respectively). Further EC_50_ analysis is in the online supplement.

## Discussion

We observed that Nrf2 and Keap-1 protein and gene expression were similar in COPD alveolar macrophages and bronchial epithelium compared to controls. However, there was strong evidence that Nrf2 associated anti-oxidant pathways were dysregulated in alveolar macrophages; NQO1 activity was downregulated, while NQO1, HMOX1, SOD1 and TXNRD1 gene expression were increased in COPD patients compared to controls. Despite similar Nrf2 expression in COPD compared to controls, these findings indicate significant downstream dysregulation of the Nrf2 pathway in COPD lung cells.

A range of Nrf2 activator compounds increased NQO1 activity, and upregulated anti-oxidant gene expression, with greater upregulation for NQO1 and TXNRD1 relative to HMOX1 and SOD1. The PPI inhibitors GSK compound 7 and C4X_6665 showed similar maximal effects to electrophilic compounds for NQO1 upregulation. The potency (assessed by calculating EC_50_ values) of C4X_6665 on NQO1 activity and regulation of anti-oxidant gene expression was higher than other Nrf2 activators. The potency of C4X_6665 shown here in assays using cells from COPD patients suggests the therapeutic potential of this novel compound to modulate dysregulated Nrf2 pathways in COPD.

PPI inhibitors bind non-covalently to Keap-1 to prevent Nrf2 binding (Abed et al. 2015). An advantage of PPI inhibitors is increased target specificity compared to electrophilic compounds which activate Nrf2 through covalent modification of cysteine residues on Keap-1, but also have the ability to modify cysteine thiols in many other molecules. The off-target binding of electrophilic inhibitors may act on multiple cellular pathways (de Zeeuw et al. 2013) resulting in an increased propensity for off-target side effects. The selectivity offered by direct, non-covalent antagonism of the Keap-1-Nrf2 interaction by PPI inhibitors may therefore have the potential for a larger therapeutic index (Davies et al. 2016). We observed some instances of enhanced gene expression modulation at high concentrations of electrophilic compounds which may be due to Nrf2-independent effects, for example the HMOX1 response to CDDO, and the SOD1 and TXNRD1 responses to Sulforaphane.

Animal models and in-vitro experiments have demonstrated beneficial efficacy for Nrf2 activator compounds on inflammation and anti-oxidant responses (Strulovici-Barel et al. 2010; Linker et al. 2011; Pergola et al. 2011; Gold et al. 2012; Sussan et al. 2009). Furthermore, pharmacological activation of Nrf2 can delay the progression of experimental emphysema (Rangasamy et al. 2004). The PPI inhibitor GSK compound 7 protects human lung epithelial cells from oxidative stress-mediated responses (Davies et al. 2016). Here, we show further evidence that PPI inhibitors, namely C4X_6665 and GSK compound 7, can modulate the activity of anti-oxidant pathways relevant to COPD. However, the oral administration of Sulforaphane in clinical trials in COPD patients and in asthma patients (using broccoli sprout extract source of Sulforaphane) failed to increase antioxidant genes or reduce oxidative stress. This may be due, at least partly, to the low potency of Sulforaphane, demonstrated here and in other studies (Bewley et al. 2018). C4X_6665 had the lowest EC_50_ concentration in our studies, followed by GSK compound 7; these had approximately ten and threefold higher potency (respectively) than CDDO for NQO1 activity. The target-specific medicinal chemistry design of the PPI inhibitors has enabled the discovery of higher potency molecules, as observed here.

We observed differential effects of Nrf2 activators on anti-oxidant gene expression, with greater effects on NQO1 and TXNRD1 expression relative to SOD1 and HMOX1. Under basal conditions, in addition to the Keap-1/Nrf2 complex, anti-oxidant gene activation is also regulated via the ARE by the transcriptional repressor Bach1 (Dhakshinamoorthy et al. 2005; Sun et al. 2002). Bach1 competes with Nrf2 in the regulation of the ARE (Dhakshinamoorthy et al. 2005). Due to differences in the presence of ARE enhancer sites at different anti-oxidant genes, the dynamic interplay between Bach1 and Nrf2 can produce distinct regulatory expression (Reichard et al. 2007). Specifically, Bach1 removal is necessary for Nrf2 mediated HMOX1 but not TXNRD1 expression (Reichard et al. 2007), which may explain the differences in Nrf2 activator effects observed here. Bach1 expression along with Keap-1 has been shown to be increased in lung tissue and alveolar macrophages in patients with severe emphysema (Goven et al. 2008) suggesting increased repression of anti-oxidant genes in COPD.

Lower NQO1 baseline activity was observed in COPD compared to control (smokers) alveolar macrophages. However, there were no differences in the effect size (fold increase) in NQO1 activity for any compound when comparing the effects between COPD and control cells. This indicates that the effects of these compounds are neither reduced or enhanced in COPD versus smoker`s macrophages.

Nrf2 activation in macrophages by Sulforaphane has been shown to increase bacterial phagocytosis and alter macrophage phenotype (Bewley et al. 2018; Harvey et al. 2011). While not investigated here, it would be valuable to further investigate the effects of PPI inhibitors on macrophage phenotype, inflammatory responses and phagocytic function, as the bacterial infection is a common problem in COPD patients, and there is evidence that COPD macrophages display reduced phagocytosis ability (Taylor et al. 2010).

Nrf2 target genes have been shown to be upregulated in epithelia and macrophages of healthy smokers (Hubner et al. 2009). Also NFE2L2 mRNA expression and its downstream targets are increased in epithelial cells isolated from COPD current smokers compared to COPD former smokers (Sidhaye et al. 2019) and in PBMCs from mild COPD patients (Fratta Pasini et al. 2016, 2020). However other groups observed decreased expression of NFE2L2 mRNA in bronchial epithelium and alveolar macrophages from mild COPD patients compared to controls (Suzuki et al. 2008) and decreased NFE2L2 and increased KEAP1 expression in the whole lung and alveolar macrophages from COPD patients with severe emphysema (average FEV1 22%) (Goven et al. 2008). Here, we studied protein expression and activity as well as gene expression. Overall, we observed no differences in epithelial expression or alveolar macrophage expression of Nrf2 or Keap-1 in COPD patients compared to smoking and non-smoking controls. However, the absolute number of alveolar macrophages expressing Nrf2 and Keap-1 were greater in COPD. In contrast, we observed significant dysregulation of the downstream Nrf2 pathway.

The reasons for differences between studies with regard Nrf2 expression may relate to disease severity. A longitudinal study showed that lower expression of Nrf2 and dependent genes were associated with a greater decline in FEV_1_ (Fratta Pasini et al. 2020), indicating that this pathway shows heterogeneous activity levels within COPD cohorts. Reduced Nrf2 expression may, therefore, be more prominent in more severe COPD, and our study had mainly moderate COPD patients (mean FEV1 68% predicted). Furthermore, the expression and function of Nrf2 are likely to be under dynamic physiological control in COPD patients driven by various factors including oxidative stress burden. This was seen to be the case in PBMCs from patients with mild-moderate COPD which showed increased Nrf2 expression under oxidative stress (Fratta Pasini et al. 2016).

There may also be an important contribution of different analytical methods of Nrf2 detection to the divergent results seen between studies with regards to COPD patients versus controls. We used immunohistochemistry for protein detection, while other studies have used Western blot and immunofluorescence. Despite no difference in Nrf2 / Keap-1 protein or gene expression in alveolar macrophages, we showed an increase in the expression of classic Nrf2 target proteins NQO1 and HMOX1 and other anti-oxidants SOD1 and TXNRD1 in alveolar macrophages from COPD patients. These data add to the growing evidence of Nrf2 signalling dysregulation playing a role in COPD.

In summary, we have shown Nrf2 and Keap-1 expression profiles in both alveolar macrophages and bronchial epithelium to be similar in samples derived from COPD patients compared to samples from controls. However, there was dysregulation of the Nrf2 signalling pathway in alveolar macrophages from COPD patients. Nrf2 activator compounds were able to induce Nrf2 target genes in alveolar macrophages from COPD patients. Moreover, the novel PPI Nrf2 compound C4X_6665 showed higher potency, in inducing NQO1 activity and upregulating anti-oxidant gene expression in these cells than comparator PPI and electrophilic Nrf2 activators. These results highlight the potential for PPI inhibitors to address Nrf2 pathway dysregulation in COPD macrophages.

## Supplementary Information

Below is the link to the electronic supplementary material.

Supplementary file1 (DOCX 36 KB)

Supplementary file2 (TIF 553 KB)

Supplementary file3 (TIF 602 KB)

Supplementary file4 (JPG 1229 KB)

Supplementary file5 (JPG 102 KB)

Supplementary file6 (JPG 107 KB)

Supplementary file7 (TIF 1091 KB)

Supplementary file8 (TIF 1028 KB)

Supplementary file9 (TIF 487 KB)

Supplementary file9 (TIF 1155 KB)

## Data Availability

The datasets used and/or analysed during the current study are available from the corresponding author on reasonable request.
