# Supplementary material for: COPD lung studies of Nrf2 expression and the effects of Nrf2 activators
Source: Inflammopharmacology. 2022 Apr 20;30(4):1431–43. doi: 10.1007/s10787-022-00967-3 (PMC9293829; doi:10.1007/s10787-022-00967-3)
Supplement: Supplementary file 1 — Supplementary file1 (DOCX 36 KB) [file 10787_2022_967_MOESM1_ESM.docx]

**COPD lung studies of Nrf2 expression and the effects of Nrf2 activators**

Jian Li^1^, James Baker^1^, Andrew Higham^1^, Rajesh Shah^2^, Angeles Montero-Fernandez^3^, Clare Murray^4^, Nicky Cooper^4^, Cathy Lucas^4^, Craig Fox^4^, Dave Singh^1,5^ and Simon Lea^1^

1. Division of Infection, Immunity and Respiratory Medicine, School of Biological Sciences,

Faculty of Biology, Medicine and Health, Manchester Academic Health Science Centre, The

University of Manchester and Manchester University NHS Foundation Trust, Manchester, UK.

2. Department of Thoracic Surgery, Manchester University Hospital NHS Foundation Trust, Manchester, UK.

3. Department of Histopathology, Manchester University Hospital NHS Foundation Trust, Manchester, UK

4. C4X Discovery Ltd, Manchester, UK

5. Medicines Evaluation Unit, The Langley Building, Southmoor Road, Manchester University Hospital NHS Foundation Trust, Manchester, UK

**Corresponding author**

Dr Simon Lea

2^nd^ Floor Office Education and Research Centre

Wythenshawe Hospital

Southmoor Road

M23 9LT

Simon.lea@manchester.ac.uk

**Results**

**Effect of Nrf2 activator compounds on alveolar macrophage NQO1 expression and NQO1 activity**

The maximal levels of NQO1 expression reached were greater in COPD compared to S (Supplement Figure 8). However, the unstimulated (basal) levels of NQO1 expression were also greater in COPD compared to S (Figure 3).

The opposite was observed for NQO1 activity with the maximal levels of NQO1 activity reached lower in alveolar macrophages from COPD compared to S (Supplement Figure 9) reaching significance for GSK7, C4X_6665 and Sulforaphane (Supplement Figure 9). Again unstimulated (basal) levels of NQO1 activity were lower before compound treatment in COPD (Figure 3).

**Effect of Nrf2 activators on HMOX1, SOD1 and TXNRD1 expression in alveolar macrophages**

EC_50_ values for HMOX1, SOD1 and TXNRD1 mRNA expression in both S and COPD patients could be calculated for CDDO, GSK7 and C4X_6665, with the dilution series of the other drugs too small to allow this calculation (Supplement Table S5). For HMOX1, SOD1 and TXNRD1 C4X_6665 had the lowest EC_50_ values.

Maximal effects on HMOX1, SOD1 and TXNRD1 expression were similar between S and COPD groups for each compound. The maximal levels of HMOX1, SOD1 and TXNRD1 expression reached were greater in alveolar macrophages from COPD compared to S.

**Table S1 Demographics of the study population (IHC)**

|  | **Never Smoker** | **Smoker** | **COPD** | **ANOVA p value** |
| --- | --- | --- | --- | --- |
| **n** | 12 | 12 | 12 | N/A |
| **Age (y)** | 67 (11) | 63 (5) | 64 (8) | p>0.05 |
| **Gender: male (%)** | 25 | 58 | 50 | p>0.05 |
| **FEV_1_ (L)** | 2.2 (0.7) | 2.4 (0.4) | 2.0 (0.4) | p>0.05 |
| **FEV_1_ % predicted** | 102 (14) | 99 (17) | 71 (18)*ꝉ | p=0.001 |
| **FVC (L)** | 2.7 (0.9) | 3.3 (0.7) | 3.2 (0.6) | p>0.05 |
| **FEV_1_/FVC Ratio (%)** | 83 (7.8) | 73 (4.2)¶ | 57 (11) *ꝉ | p<0.0001 |
| **Current smokers (%)** | N/A | 100 | 100 | p>0.05 |
| **Pack year history** | N/A | 44 (16) | 52 (24) | p>0.05 |
| **ICS users (%)** | N/A | N/A | 17 | N/A |
| **LABA users (%)** | N/A | N/A | 17 | N/A |
| **LAMA users (%)** | N/A | N/A | 25 | N/A |

**FEV_1_ forced expiratory volume in 1 second; FVC forced vital capacity; ICS inhaled corticosteroids. LABA long-acting beta-2 agonists; LAMA long-acting muscarinic antagonists Data presented as mean (SD). Tukey’s multiple comparisons test performed where ¶ = significant difference between NS and S; * = significant difference between COPD vs NS; ꝉ = significant difference between COPD vs S.**

**Table S2 Demographics of the study population for alveolar macrophage baseline NFE2L2/KEAP1 mRNA expression**

|  | **Never Smoker Smoker** | | **COPD** | **p value** |
| --- | --- | --- | --- | --- |
| **n** | 8 | 25 | 29 | N/A |
| **Age** | 71 (13) | 73 (7) | 68 (8) | p>0.05 |
| **Gender: male (%)** | 13 | 36 | 31 | p>0.05 |
| **FEV1 (L)** | 2.5 (0.1) | 2.2 (0.5) | 1.6 (0.4) *ꝉ | p<0.0001 |
| **FEV_1_ % predicted** | 126 (24) | 101 (21) | 70 (13) *ꝉ | p<0.0001 |
| **FVC (L)** | 3.24 (1.1) | 2.98 (0.7) | 2.95 (0.8) | p>0.05 |
| **FEV_1_/FVC ratio (%)** | 83 (9) | 80 (14) | 56 (9) *ꝉ | p<0.0001 |
| **Current smokers (%)** | N/A | 52 | 61 | p>0.05 |
| **Pack-year history** | N/A | 36 (13) | 52 (45) | p>0.05 |
| **ICS users (%)** | N/A | N/A | 14 | N/A |
| **LABA users (%)** | N/A | N/A | 35 | N/A |
| **LAMA users (%)** | N/A | N/A | 17 | N/A |

**FEV_1_ forced expiratory volume in 1 second; FVC forced vital capacity; ICS inhaled corticosteroids. LABA long-acting beta-2 agonists; LAMA long-acting muscarinic antagonists Data presented as mean (sd). Tukey’s multiple comparisons test performed where * = significant difference between COPD vs NS; ꝉ = significant difference between COPD vs S.**

**Table S3 Demographics of the population for alveolar macrophages culture**

| **Clinical characteristics** | **S COPD** | | **P value** |
| --- | --- | --- | --- |
| **n** | 13 | 17 | N/A |
| **Age (y)** | 63 (9) | 70 (7) | p>0.05 |
| **Gender: male (%)** | 30 | 47 | p>0.05 |
| **FEV1 (L)** | 2.3 (0.4) | 1.6 (0.5) | p<0.001 |
| **FEV1% predicted** | 93.8 (18.8) | 66.3 (16.5) | p<0.001 |
| **FVC (L)** | 3.1 (0.5) | 3.2 (0.7) | p>0.05 |
| **FEV1/FVC ratio (%)** | 79.1 (6.5) | 52.1 (11.9) | p*<*0.001 |
| **Current smokers (%)** | 100 | 47 | N/A |
| **Pack-year history** | 46(17) | 48(21) | p>0.05 |
| **ICS users (%)** | N | 53% | N/A |
| **LABA users (%)** | N | 53% | N/A |
| **LAMA users (%)** | 8% | 47% | N/A |

**FEV_1_ forced expiratory volume in 1 second; FVC forced vital capacity; ICS inhaled corticosteroids. LABA long-acting beta-2 agonists; LAMA long-acting muscarinic antagonists Data presented as mean (SD). Tukey’s multiple comparisons test performed significant difference between COPD vs S.**

**Table S4 Comparisons of CDDO, GSK7 and C4X_6665 effects on alveolar macrophage NQO1 activity and NQO1 mRNA expression at matched concentrations**

|  | **Effect (fold increase)** | | | | | |
| --- | --- | --- | --- | --- | --- | --- |
|  | **S (N=8)** | | | **COPD (N=8)** | | |
|  | **CDDO** | **GSK7** | **C4X_6665** | **CDDO** | **GSK7** | **C4X_6665** |
| **NQO1 Activity** |  |  |  |  |  |  |
| 30 nM  10 nM  3 nM  1 nM | 1.70  1.40  1.14  1.13 | 1.64  1.48  1.26  1.17 | 1.78 **  1.76 ** **^###^**  1.53 ** **^#^**  1.35 ** | 1.90  1.69  1.39  1.39 | 1.67  1.55  1.33  1.23 | 1.82  1.73**  1.66 **^#^**  1.47 |
| **NQO1 mRNA expression** |  |  |  |  |  |  |
| 30 nM | 2.47 | 2.51 | 2.82 | 2.88 | 2.09 | 2.61 |
| 10 nM  3 nM  1 nM | 1.63  1.33  1.14 | 1.89  1.45  1.17 | 2.65 ** ^###^  2.23 ** ^##^  1.60 | 1.82  1.54  1.45 | 1.68  1.36  1.20 | 2.54 * ^#^  2.22 ** ^###^  1.61 |

***, ** = significantly above GSK7 (p<0.05, 0.01)**

**^#, ##, ###^ = significantly above CDDO (p<0.05, 0.01, 0.001)**

**Table S5 Maximum effect and EC50s of Nrf2 activator compounds on alveolar macrophage HMOX1, SOD1 and TXNRD1 mRNA expression**

|  | **S (N=8)^#^** | | **COPD (N=8)** | |
| --- | --- | --- | --- | --- |
|  | **Maximum effect (fold increase)** | **EC_50_ (nM)** | **Maximum effect (fold increase)** | **EC_50_ (nM)** |
| **HMOX1mRNA expression** |  |  |  |  |
| CDDO | 1.99 | 26.6 | 3.73 ^†^ | 37.8 |
| GSK Compound 7 | 1.70 | 16.7 | 1.46 | 7.2 |
| C4X_6665  Sulforaphane  MMF  ANOVA p value | 1.28  1.73  1.41  0.074 | 2.7  -  - | 1.37  1.49  1.21  0.0086 | 1.4  -  - |
| **SOD1 mRNA expression** |  |  |  |  |
| CDDO | 1.70 ^††^ | 10.3 | 1.53 ^†^ | 4.8 |
| GSK Compound 7 | 1.31 | 32.8 | 1.25 | 31.9 |
| C4X_6665  Sulforaphane  MMF  ANOVA p value | 1.35  2.55 **^¶¶ *^** **^$^** ^†^  1.16  0.0027 | 3.2  -  - | 1.34 ^††^  2.52 ^†^  1.10  0.0058 | 3.1  -  - |
| **TXNRD1 mRNA expression** |  |  |  |  |
| CDDO | 3.33 ^†^ | 9.8 | 2.30 ^††^ | 5.7 |
| GSK Compound 7 | 3.78 ^††^ | 26.4 | 2.85 **^¶^** ^††^ | 30.4 |
| C4X_6665  Sulforaphane  MMF  ANOVA p value | 3.37 ^††^  5.14 **^¶¶^** **^$^** ^††^  1.68  <0.0001 | 1.6  -  - | 2.70 ^†††^  4.28 **^¶¶^** *** **^$^** ^†††^  1.46  <0.0001 | 2.0  -  - |

**# Data from 8 smokers for HMOX1and 6 smokers for SOD1 and TXNRD1**

***, ** = significantly above GSK7 (p<0.05, 0.01)**

**^¶, ¶¶^ = significantly above CDDO (p<0.05, 0.01)**

**^$, $$^ = significantly above C4X_6665 (p<0.05, 0.01)**

**^†, ††, †††^ = significantly above MMF (p<0.05, 0.01, 0.001)**

**Supplement Figure 1**

**Effect of Nrf2 activator compounds on NQO1 mRNA in BEAS-2B bronchial epithelial cell line.**

BEAS-2B cells (ATCC) were treated with CDDO, GSK7, C4X_6665, Sulforaphane, MMF or vehicle control (DMSO) for 24h. NQO1 mRNA expression was assessed by RT-qPCR. Data presented as mean fold increase above DMSO control. Four parameter non-linear iterative curve fitting analysis for individual compounds was used to generate curves.

**Supplement Figure 2**

**Effect of Nrf2 activator compounds on alveolar macrophage cell viability.** Alveolar macrophages from S (n=8) and COPD patients (n=8) were treated with CDDO (100 nM), GSK7 (1000nM), C4X_6665 (30 nM), Sulforaphane (10000nM), MMF (10000nM) or vehicle control (DMSO) for 48 h. Cell viability was assessed by lactate LDH assay. Data shown for the highest concentrations tested.

**Supplement Figure 3**

**Representative images of isolated alveolar macrophages post cultured**

Alveolar macrophages were treated with vehicle control (DMSO) (A), CDDO (100 nM) (B), GSK7 (1000nM) (C), C4X_6665 (30 nM) (D), Sulforaphane (10000nM) (E) or MMF (10000nM) (F) for 24 hours. Cells were stained with haemotoxylin and eosin and imaged.

**Supplement Figure 4**

**Expression** **of** **Nrf2 and Keap-1 in the alveolar macrophages of NS, S and COPD patients.** Protein expression of Nrf2 and Keap-1 was examined by immunohistochemistry in lung tissue from NS (n=12), S (n=12) and COPD (n=12). Representative images show alveolar macrophages positive for Nrf2 (A,C,E) and Keap-1 (B,D,F) in NS (A-B), S (C-D) and COPD patients (E-F). Black arrows indicate alveolar macrophages positive for Nrf2 or Keap-1 staining, red arrows indicate alveolar macrophages negative for Nrf2 or Keap-1 staining.

**Supplement Figure 5**

**Expression of** **Nrf2 and Keap-1 in the bronchial epithelium of NS, S and COPD patients.**

In a subset of patients with small airways present on the stained section protein expression of Nrf2 and Keap-1 was examined by immunohistochemistry in lung tissue from NS (n=9), S (n=12) and COPD (n=8). Representative images show bronchial epithelium positive for Nrf2 (A,C,E) and Keap-1 (B,D,F) in NS (A-B), S (C-D) and COPD patients (E-F). Black arrows indicate bronchial epithelium positive for Nrf2 or Keap-1 staining, red arrows indicate alveolar macrophages negative for Nrf2 or Keap-1 staining.

**Supplement Figure 6**

**Expression of** **Nrf2 and Keap-1 in the alveolar macrophages of COPD patients.** Protein expression of Nrf2 (A) and Keap-1 (B) was examined by immunohistochemistry in lung tissue from COPD patients on no treatment (n=7) or those patients taking inhaled corticosteroids and / or long acting bronchodilator treatment (≥1 ICS/LABA/LAMA) (n=5). Data presented as percentage of positive cells of alveolar macrophages (AM) for Nrf2 (A) and Keap-1 (B). mRNA expression levels of NFE2L2 (D) and KEAP1 (E) were determined by RT-qPCR in AM from COPD patients on no treatment (n=18) or ≥1 ICS/LABA/LAMA (n=11). Data represents individual patients with mean. RT-qPCR data expression relative to endogenous control (2^−Δ^C_t_).

**Supplement Figure 7**

**Expression** **of** **Nrf2 and Keap-1 in the bronchial epithelium COPD patients.** Protein expression of Nrf2 and Keap-1 was examined by immunohistochemistry in lung tissue from COPD patients on no treatment (n=4) or those patients taking inhaled corticosteroids and / or long acting bronchodilator treatment (≥1 ICS/LABA/LAMA) (n=4). Data presented as positive cells per mm of epithelium for Nrf2 (A) and Keap-1 (B). Data represented as positive cells intensity of epithelium for Nrf2 (C) and Keap-1 (D). Data represents individual patients with mean.

**Supplement Figure 8**

**Effect of Nrf2 activator compounds on NQO1 mRNA in alveolar macrophage from S and COPD patients.**

Alveolar macrophages from S (n=8) and COPD patients (n=8) were treated with CDDO (0.3–100 nM) (A), GSK7 (1-1000nM) (B), C4X_6665 (0.3-30 nM) (C), Sulforaphane (1000-10000nM) (D), MMF (1000-10000nM) (E) or vehicle control (DMSO) for 24 h. NQO1 mRNA expression was assessed by RT-qPCR. Data presented as mean ± SEM NQO1 mRNA expression levels relative to endogenous control (2^−Δ^C_t_).

*, **, *** = significant difference between groups for matched drug concentration (p<0.05, p<0.01 and p<0.001 respectively).

**Supplement Figure 9**

**Effect of Nrf2 activator compounds on NQO1 activity in alveolar macrophage from S and COPD patients.**

Alveolar macrophages from S (n=8) and COPD patients (n=8) were treated with CDDO (0.3–100 nM) (A), GSK7 (1-1000nM) (B), C4X_6665 (0.3-30 nM) (C), Sulforaphane (1000-10000nM) (D), MMF (1000-10000nM) (E) or vehicle control (DMSO) for 48 h. NQO1 activity was assessed by NQO1 activity enzyme assay. Data presented as mean ± SEM absolute NQO1 activity (change in absorbance 0-30 minutes at 570nm).

*, **, *** = significant difference between groups for matched drug concentration (p<0.05, p<0.01 and p<0.001 respectively).
