# Supplementary figures and images for: COPD lung studies of Nrf2 expression and the effects of Nrf2 activators
Source: Inflammopharmacology. 2022 Apr 20;30(4):1431–43. doi: 10.1007/s10787-022-00967-3 (PMC9293829; doi:10.1007/s10787-022-00967-3)

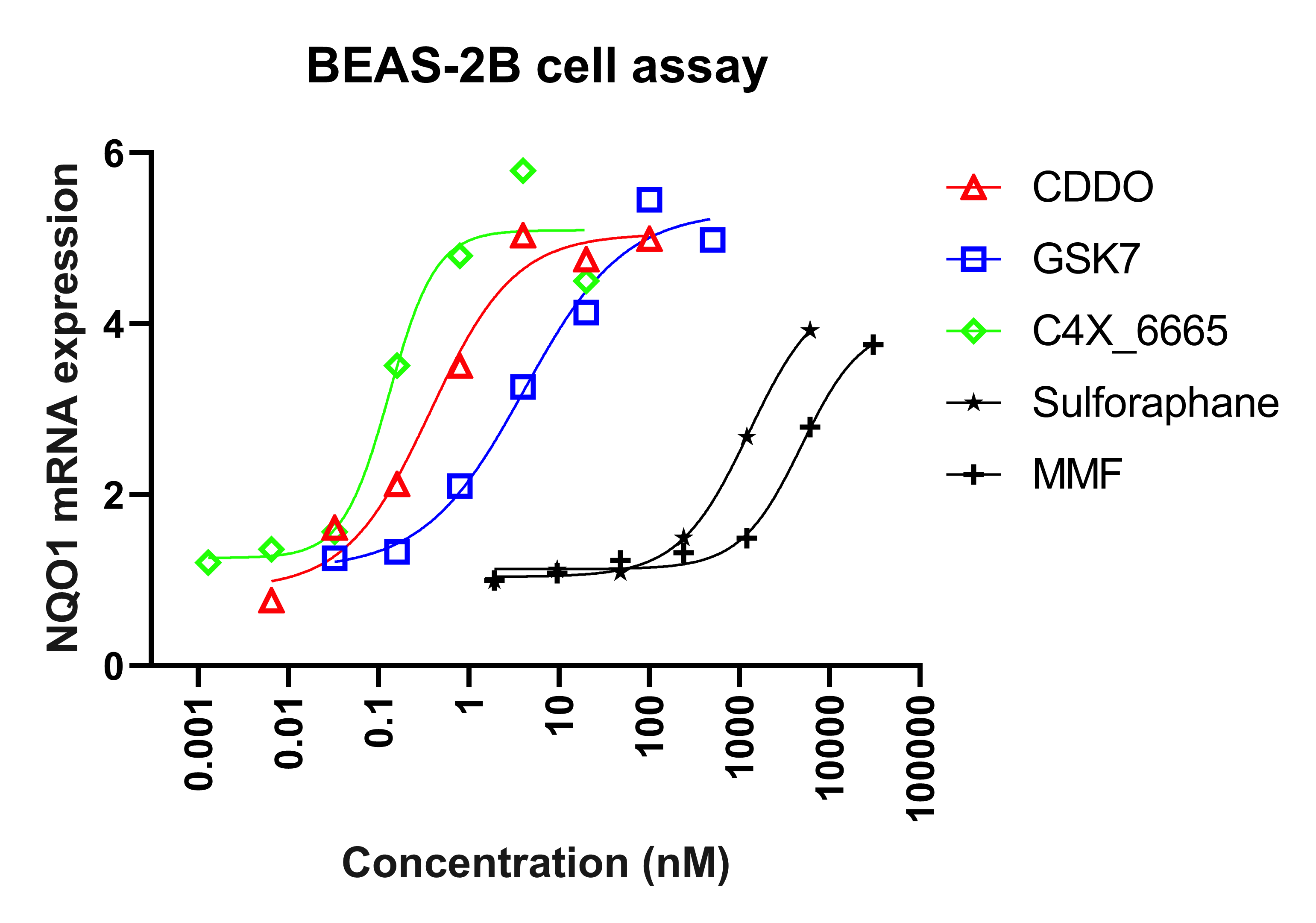

Supplement: Supplementary file 2 — Supplementary file2 (TIF 553 KB) [file 10787_2022_967_MOESM2_ESM.tif]

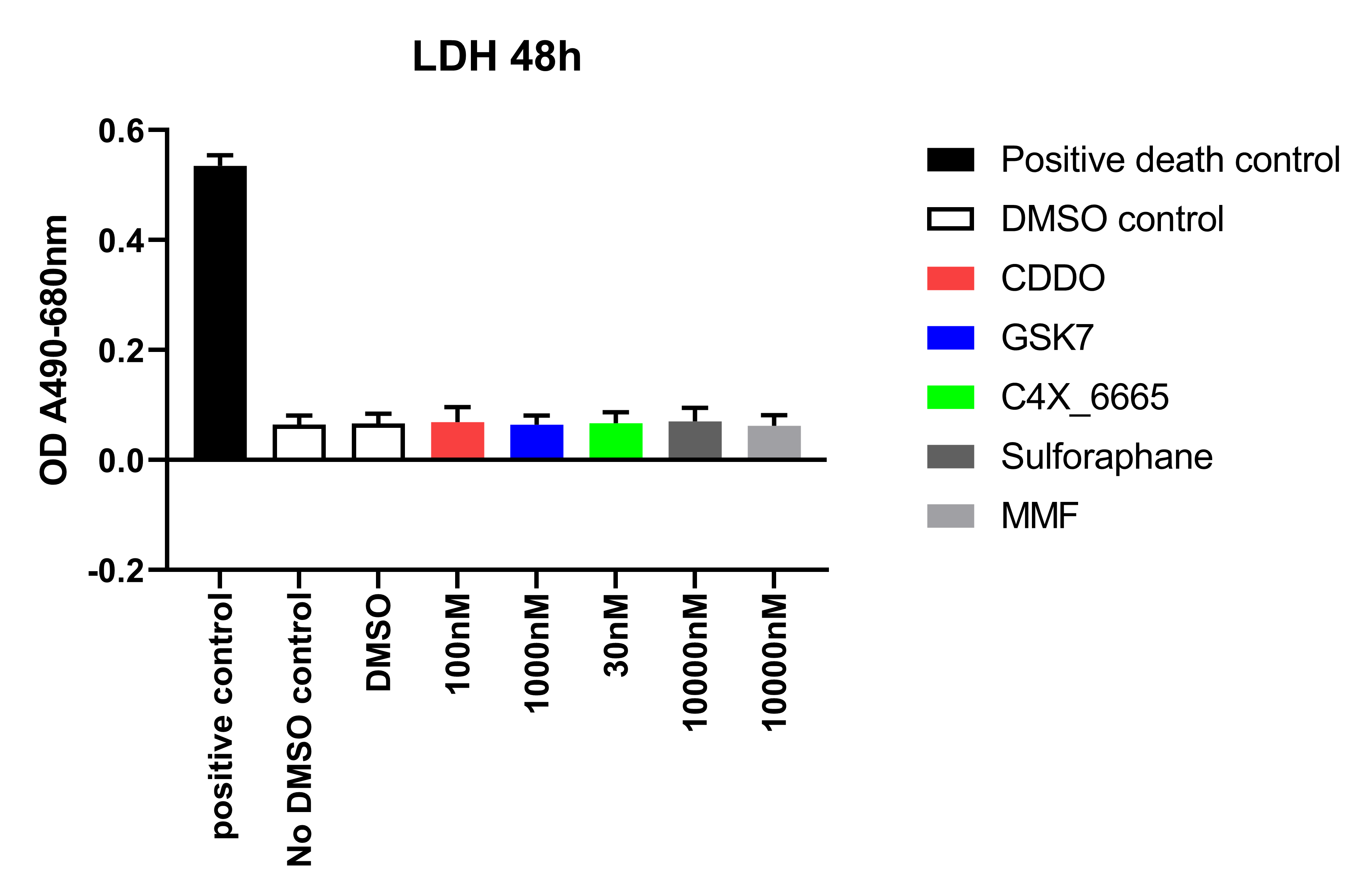

Supplement: Supplementary file 3 — Supplementary file3 (TIF 602 KB) [file 10787_2022_967_MOESM3_ESM.tif]

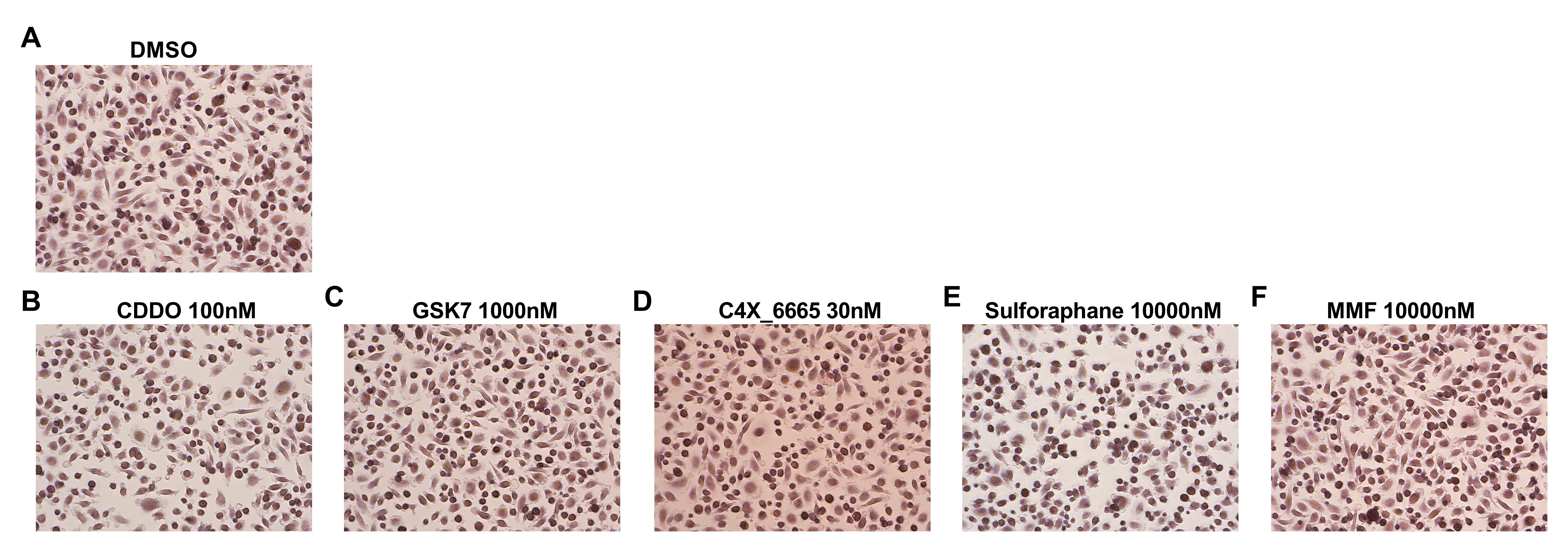

Supplement: Supplementary file 4 — Supplementary file4 (JPG 1229 KB) [file 10787_2022_967_MOESM4_ESM.jpg]

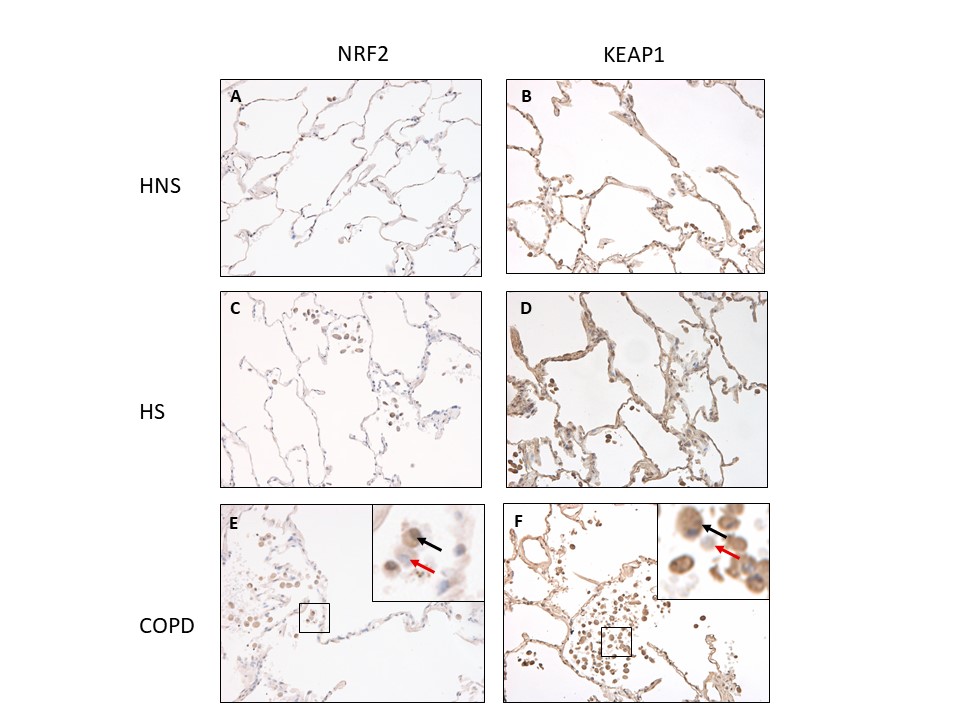

Supplement: Supplementary file 5 — Supplementary file5 (JPG 102 KB) [file 10787_2022_967_MOESM5_ESM.jpg]

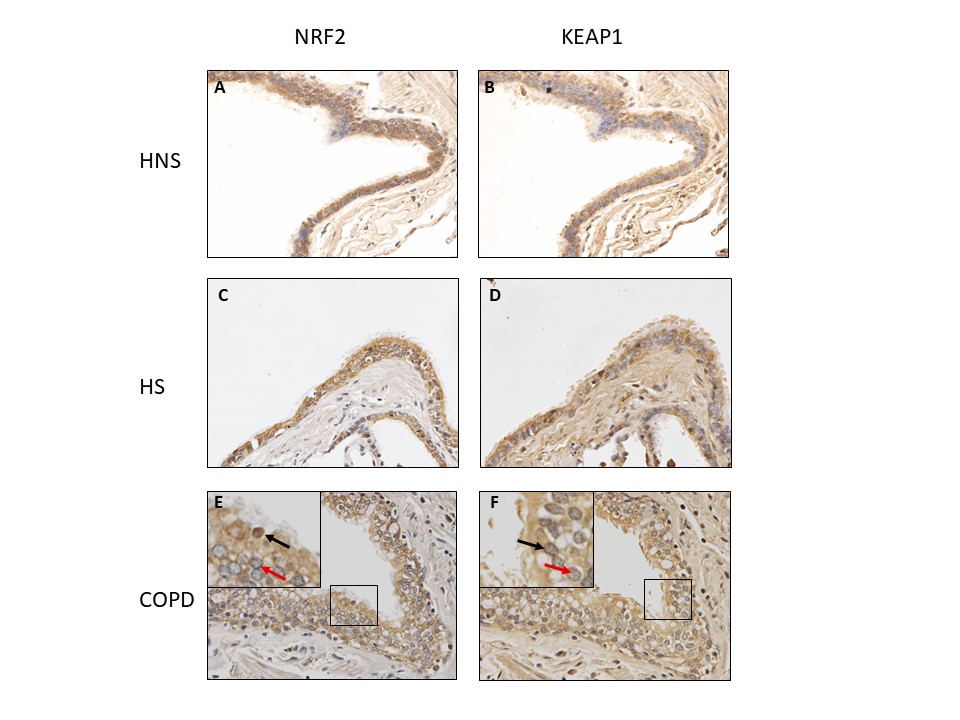

Supplement: Supplementary file 6 — Supplementary file6 (JPG 107 KB) [file 10787_2022_967_MOESM6_ESM.jpg]

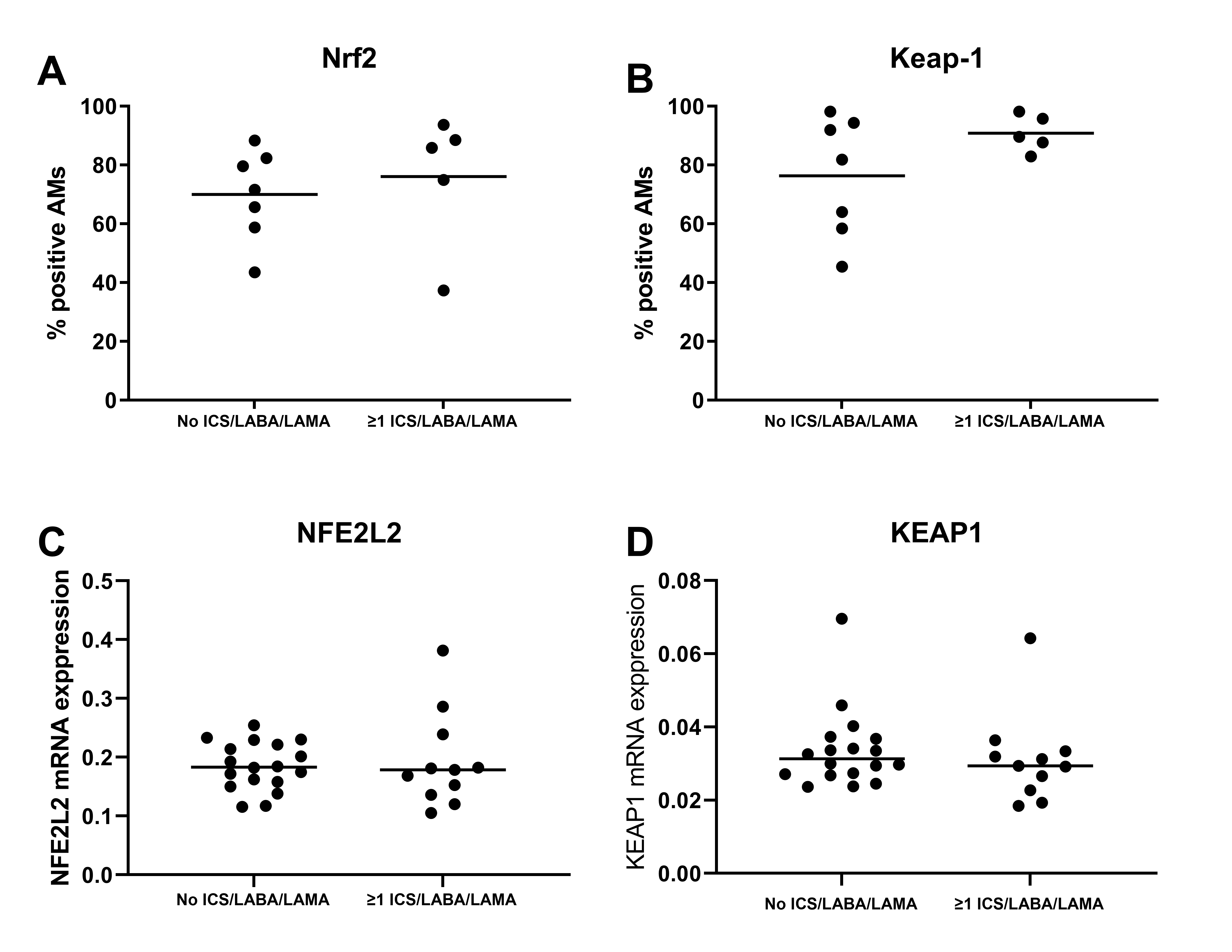

Supplement: Supplementary file 7 — Supplementary file7 (TIF 1091 KB) [file 10787_2022_967_MOESM7_ESM.tif]

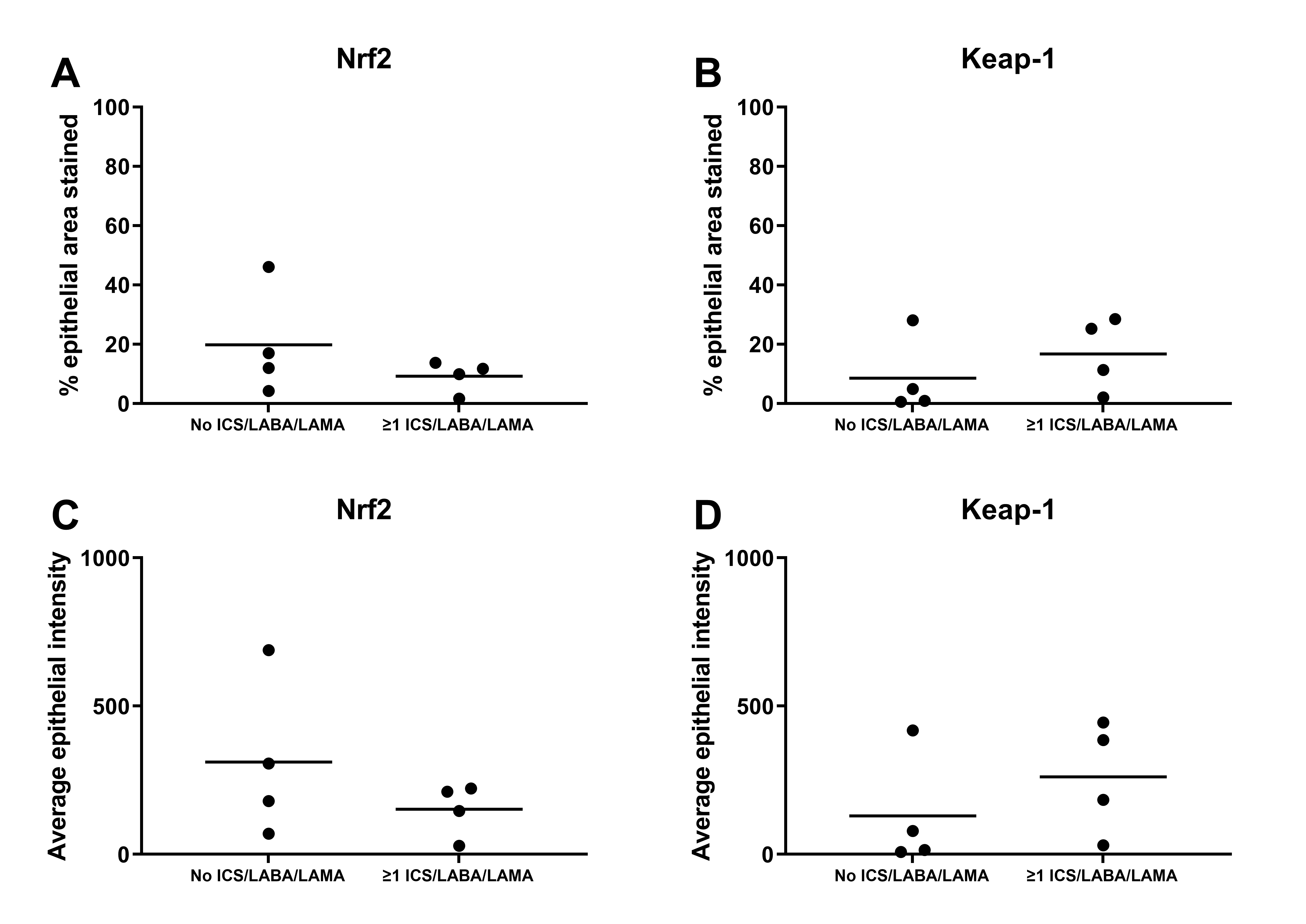

Supplement: Supplementary file 8 — Supplementary file8 (TIF 1028 KB) [file 10787_2022_967_MOESM8_ESM.tif]

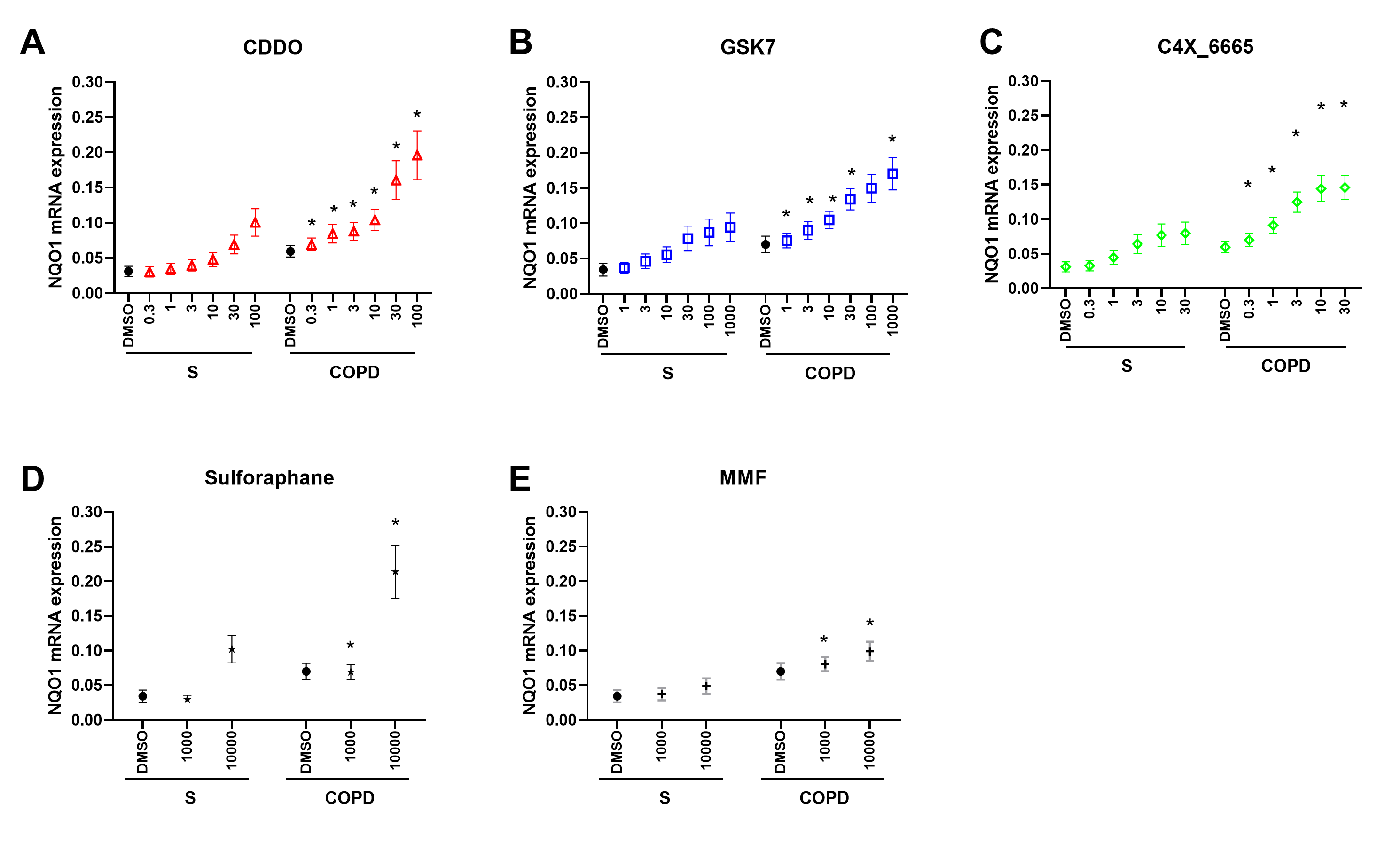

Supplement: Supplementary file 9 — Supplementary file9 (TIF 487 KB) [file 10787_2022_967_MOESM9_ESM.tif]

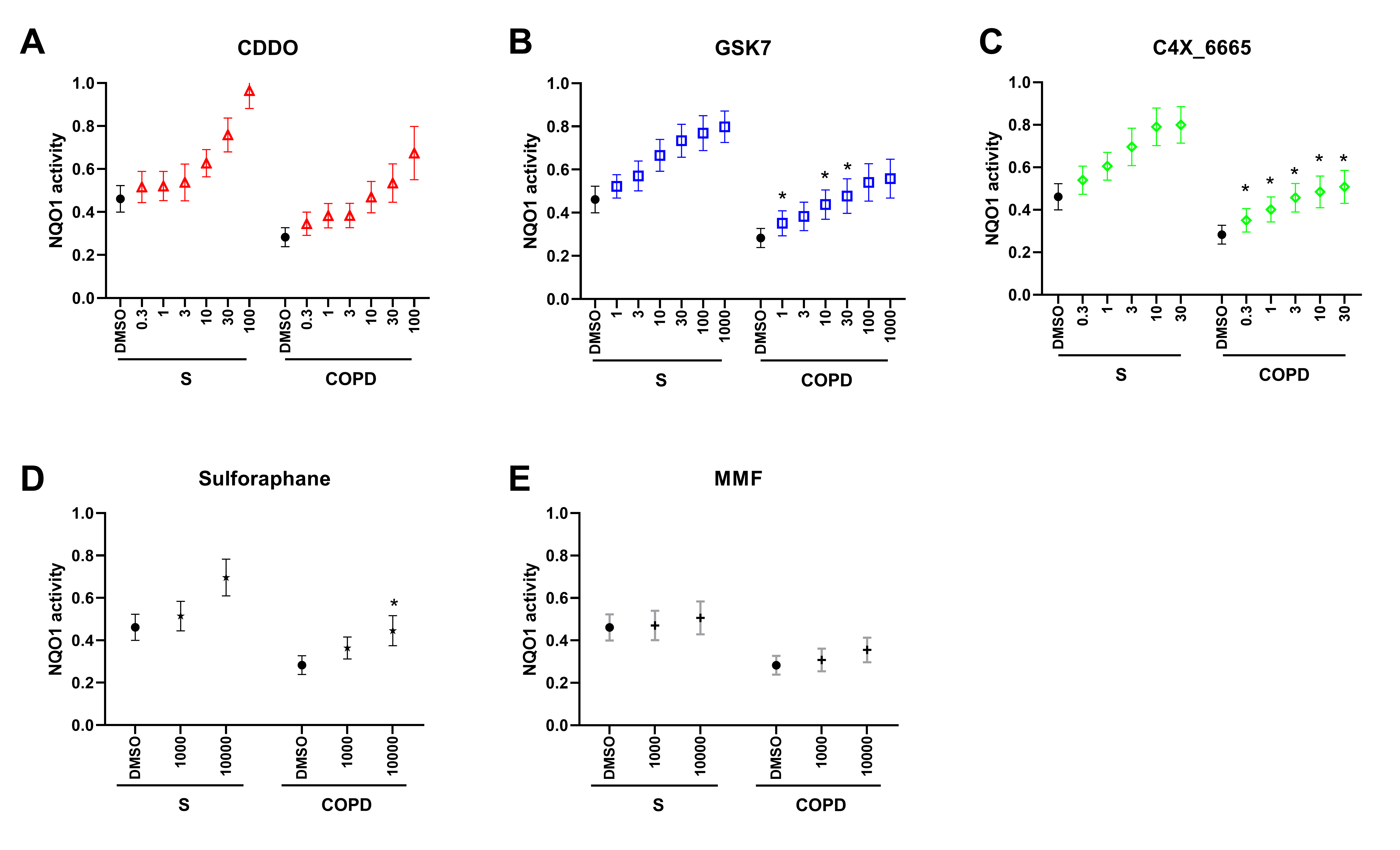

Supplement: Supplementary file 10 — Supplementary file9 (TIF 1155 KB) [file 10787_2022_967_MOESM10_ESM.tif]
